# Semantic Processing in Deaf and Hard-of-Hearing Children: Large N400 Mismatch Effects in Brain Responses, Despite Poor Semantic Ability

**DOI:** 10.3389/fpsyg.2016.01146

**Published:** 2016-08-10

**Authors:** Petter Kallioinen, Jonas Olofsson, Cecilia Nakeva von Mentzer, Magnus Lindgren, Marianne Ors, Birgitta S. Sahlén, Björn Lyxell, Elisabet Engström, Inger Uhlén

**Affiliations:** ^1^Department of Linguistics, Stockholm UniversityStockholm, Sweden; ^2^Lund University Cognitive Science, Lund UniversityLund, Sweden; ^3^Department of Psychology, Stockholm UniversityStockholm, Sweden; ^4^Department of Behavioral Sciences and Learning, Swedish Institute for Disability Research, Linkoping UniversityLinkoping, Sweden; ^5^Linneaus Centre, Cognition, Communication and Learning, Lund UniversityLund, Sweden; ^6^Department of Psychology, Lund UniversityLund, Sweden; ^7^Division of Clinical Neurophysiology, Department of Clinical Neuroscience, Lund UniversityLund, Sweden; ^8^Division of Clinical Neurophysiology, Department of Clinical Neuroscience, Skåne University HospitalLund, Sweden; ^9^Logopedics, Phoniatrics and Audiology, Department of Clinical Sciences Lund UniversityLund, Sweden; ^10^Department of Hearing and Balance, Karolinska University Hospital and Karolinska Institutet (CLINTEC)Stockholm, Sweden

**Keywords:** children, cochlear implants, hearing aids, semantics, N400 evoked potential

## Abstract

Difficulties in auditory and phonological processing affect semantic processing in speech comprehension for deaf and hard-of-hearing (DHH) children. However, little is known about brain responses related to semantic processing in this group. We investigated event-related potentials (ERPs) in DHH children with cochlear implants (CIs) and/or hearing aids (HAs), and in normally hearing controls (NH). We used a semantic priming task with spoken word primes followed by picture targets. In both DHH children and controls, cortical response differences between matching and mismatching targets revealed a typical N400 effect associated with semantic processing. Children with CI had the largest mismatch response despite poor semantic abilities overall; Children with CI also had the largest ERP differentiation between mismatch types, with small effects in within-category mismatch trials (target from same category as prime) and large effects in between-category mismatch trials (where target is from a different category than prime), compared to matching trials. Children with NH and HA had similar responses to both mismatch types. While the large and differentiated ERP responses in the CI group were unexpected and should be interpreted with caution, the results could reflect less precision in semantic processing among children with CI, or a stronger reliance on predictive processing.

## Introduction

In a spoken language environment, impaired hearing can limit the development of words, concepts and ultimately language comprehension and communication in children. Indeed, deaf and hard-of-hearing children (henceforth, DHH) children have, on average, a more limited vocabulary than their peers (e.g., Luckner and Cooke, 2010 for a review; Blamey et al., 2001; Geers et al., 2003; Le Normand et al., 2003; Kenett et al., 2013; Walker and McGregor, 2013). With a small vocabulary, an underdeveloped semantic structure (i.e., the taxonomic, associative or similarity-based relations between words) could also be expected, but research indicates a large heterogeneity among DHH children (Peterson et al., 2010; Löfkvist et al., 2012; Kenett et al., 2013; Li et al., 2013; Nakeva von Mentzer, 2014). In fact, semantic and other cognitive cues may play a more important role in linguistic processing of DHH children, as a means to compensate for poor phonological skills (Lyxell et al., 2009; Nakeva von Mentzer et al., 2014a). What cannot be extracted from the speech signal bottom-up might be inferred using top-down processes (Wingfield and Tun, 2007). Thus, predicting semantic content might be of importance for DHH persons, due to their difficulties in extracting semantic content from speech input.

Children who are DHH are characterized by widely varying etiologies and symptoms. The most common mitigation for hearing deficits in DHH children is hearing aids (HAs) and/or cochlear implants (CIs). Traditional HAs amplify sounds and optimize the auditory input for the children's residual sensory function. In contrast, CIs convert sounds to coded electrical signals that are transmitted to the auditory nerve in the cochlea, enabling access to sound. Cochlear implants have shown to recover auditory function also in profoundly deaf individuals (Henkin et al., 2003; Sullivan, 2013).

Listening through HAs or CIs is associated with specific limitations (Moore, 2008; Nittrouer et al., 2012). For example, the limited temporal and spectral resolution of the CI signal can lead to difficulties in perceiving segments in consonant clusters and other aspects of speech. Phonological skills involve the decoding of speech into linguistically relevant information such as phoneme combinations that are central for learning, storing and accessing words (Ramus and Szenkovits, 2008; Stoel-Gammon, 2010; Dillon et al., 2012). Poor phonological skills might explain DHH children's poor performance in many cognitive and linguistic tests, such as those assessing lexical access and lexical variation (Lyxell et al., 2009; Asker-Árnason et al., 2010).

The present study investigated cortical processing of semantics before and after a computer-assisted reading intervention with a phonics approach (Nakeva von Mentzer et al., 2013, 2014b). The intervention focus is on strengthening the connection between graphemes and phonemes, which was hypothesized to boost phonological awareness skills, which in turn could enhance lexical access and vocabulary development. The intervention did have effects on phonological processing, in particular for DHH children starting with low phonological skills (Nakeva von Mentzer et al., 2013). There were also effects on reading skills (Nakeva von Mentzer et al., 2014b), however semantic tasks such as lexical prediction was not affected. Children with CI performed worse than controls on auditory lexical prediction tasks (Nakeva von Mentzer et al., 2013). This is in apparent contrast to recent results using picture naming that show semantic performance et al. with controls (Löfkvist et al., 2014; Wechsler-Kashi et al., 2014), but this difference might be explained by varying difficulty in processing speech stimuli.

Semantic processing can be investigated using the event related potential (ERP) component N400, and the N400 is arguably the most studied brain response in language processing research (Kutas and Federmeier, 2011). The typical N400 component is a negative peak at centro-parietal electrodes around 400 ms after event onset, elicited by meaningful stimuli such as spoken or written words (Kutas and Federmeier, 2011), but also pictures (West and Holcomb, 2002; Franklin et al., 2007; Proverbio and Riva, 2009). Semantically improbable or incongruent stimuli elicit large negative N400 responses compared to probable or congruent stimuli. The N400 is modulated by semantic structure; when primes are semantically related, but mismatching, to targets, the N400 amplitude is reduced (Kutas and Federmeier, 2011). This relatedness effect might be due to an increased use of predictive processing (Franklin et al., 2007; Kutas and Federmeier, 2011). In study designs using picture targets, the N400 is typically preceded by the N300, a more frontal negative component that responds to very distinct semantic deviations such as unrelated or between-category mismatches (Barrett and Rugg, 1990; McPherson and Holcomb, 1999; Hamm et al., 2002).

Research on CI routinely uses ERP assessment, but the focus is often on processing of auditory stimuli in cortical auditory evoked potentials (CAPS) and auditory oddball paradigms (Groenen et al., 2001; Martin et al., 2008; Peterson et al., 2010). Traditional ERP components such as the P1-N1-P2 complex, acoustic change complex (ACC), mismatch negativity (MMN), and P3 have been used to assess auditory discrimination, maturation and intervention effects in persons with CI (Kraus et al., 1993; Okusa et al., 1999; Eggermont and Ponton, 2003; Beynon and Snik, 2004; Kral and Sharma, 2012; Näätänen et al., 2012; Timm et al., 2012; Vavatzanidis et al., 2016) and with HA (Thai-Van et al., 2010). Studies of N400 responses are scarce, in particular among DHH-children, leaving their semantic processing changes relatively unexplored on a biological level (Johnson, 2009). We are aware of only one study of N400 conducted on a child with CI. In this study (Key et al., 2010), N400 responses were recorded from a 6-year-old girl with unilateral CI from 2 years of age. Assessment before and after activating the CI resulted in a dramatic increase of the N400. A few studies report N400 results among adult CI-users with post-lingual deafness. One study (Hahne et al., 2012) assessed 13 CI-users (mean age 51 years) and found N400 effects for both semantic violations and cloze probability manipulations in an auditory sentence comprehension test. The N400 effects consisted of later and more long-lasting peaks among CI-users than controls. Another study (Finke et al., 2016) with 13 CI-users (mean age 60 years) found an N400-like effect in an oddball task with word stimuli, although the authors described it as an N2 component. Here, ERP latencies were associated with listening effort and intelligibility in the CI group. A third study (Henkin et al., 2015) assessed 9 CI-users (mean age 66 years) in a voice gender discrimination task with auditory word stimuli. Results showed nominally longer N400 latencies among CI-users compared to controls, however, the difference was not tested for statistical difference.

In the present study, we investigated semantic processing in DHH children using an N400 paradigm with spoken primes and picture targets. The spoken primes were either fully congruent with targets (matching), unrelated to the target (between-category mismatch), or a mismatching prime that was related to the target by category membership (within-category mismatch). Participating children were asked whether the picture target matched the word prime or not. The task challenged semantic processing, and allowed us to compare DHH children to matched controls with normal hearing (NH). We compared results from the two mismatch types to investigate effects related to semantic structure. We hypothesized differences in brain responses between normal hearing children (NH), children with HA and children with CI, reflecting increased semantic difficulties related to the severity of hearing impairment. Presumably this would be reflected in smaller mismatch effects overall (NH > HA > CI), or smaller response to within-category mismatches relative to between-category mismatches, due to a less fine-grained semantic structure in DHH children. We also investigated effects of a reading intervention with the phonics approach directed at beginning readers (Lovio et al., 2012). This intervention was hypothesized to strengthen phonological awareness by training grapheme-phoneme correspondence. We hypothesized that better phonological awareness among DHH children would make words more distinct and thereby easier to process semantically, resulting in larger N400 mismatch effects after training.

## Materials and methods

### Participants

This study was based on data from 42 children (21 girls) aged 5–7 years. Thirty of them were deaf or hard-of-hearing (DHH) and 12 were normal hearing controls (NH; 3 girls). Of the DHH children, 15 had bilateral hearing aids (HAs) and 15 had at least one cochlear implant (CI). In each of these groups 9 were girls. Nine children (7 girls) had bilateral cochlear implants, and six children (2 girls) had CI in one ear and hearing aid in the other. Participants were grouped based on their type of hearing amplification: NH, HA, and CI (at least one implant). Seventeen children (9 girls) had a severe/profound hearing impairment with a pure tone average (PTA) at > 70 dB Hearing Level unaided. Eleven children (7 girls) with hearing aids had a moderate HI (PTA 40-60 dB) and two children (girls) had a mild HI (PTA < 40 dB). The mean age at diagnosis was 1 year and 2 months, ranging from 0 weeks to 5 years. Seven children were diagnosed with a progressive hearing impairment, where one child was born with unilateral deafness and later developed progressive hearing impairment on the other ear. The mean age for receiving HA was 2 years and 8 months (ranging from 3 months to 6 years) and the mean age for first CI-operation was 1 year and 7 months (ranging from 11 months to 5 years). Aided thresholds with CI or HA were at 20–40 dB, with higher values in the high frequencies for children with hearing aids. Three children had another spoken language besides Swedish, two children used sign language as their first mode of communication at home and used spoken Swedish in school and two children used sign support to their spoken language. All children performed within normal limits on nonverbal intelligence as assessed by Ravens colored matrices, and there was no significant difference between the groups regarding nonverbal intelligence (see Table 1 and Nakeva von Mentzer et al., 2013). Four more children participated in the study but were excluded from the present analysis (one control did not meet inclusion criteria, one control did not participate in the training intervention, and two children with CI were excluded due to ERP-recording issues). The DHH children were found through clinical records of the participating hospitals. All children were invited who fulfilled the criteria; 5–7 years old with bilateral hearing aids and/or CIs, speaking Swedish in their educational setting, and with no known disability affecting language development. Invitations were sent to 90 families and approximately one third of those accepted to participate. The controls were recruited from preschools and schools in the Stockholm area. Written informed parental consent was obtained for all the participants. The study was approved by the Regional Committee of Medical Research Ethics in Stockholm.

**Table 1:** **Selected test results (from first ERP session) presented as means and standard deviations for each hearing amplification group**.

**Hearing amplification groups, selected test results**			
**Hearing amplification group**	**Normal hearing (NH)**	**Hearing aid (HA)**	**Cochlear implant (CI)**
	**Mean (Std)**	**Mean (Std)**	**Mean (Std)**
Age (months)	81 (12.0)	76 (11.9)	76 (11.0)
Raven colored matrices (%)	85.8 (24.0)	76.3 (18.7)	75.3 (24.5)
Phonological composite	86.7 (7.9)	68.6 (10.8)	59.3 (17.4)
Lexical access	14.8 (1.8)	13.6 (4.3)	9.3 (6.6)
Reading skill composite	0.111 (0.126)	0.044 (0.07)	0.083 (0.131)
Auditory ERP response	2.66 (0.66)	1.69 (0.76)	2.19 (1.32)
N	12	15	15

The auditory ERP response is the average amplitude at 6 fronto-central electrodes at a latency of 80–220 ms in response to both standards and 4 deviant types in a MMN paradigm (see Uhlén et al., in preparation).

### Language testing, intervention, and ERP recording

Participation started 1 month before the first ERP-recording, with a set of assessments of language and cognitive skills, conducted in a quiet room in the children's homes or in their educational setting. The same tests were repeated on the day of the first ERP recording, and on the day of the second ERP recording following a month of intervention training. Tests, scores and behavioral effects of the intervention were described previously, i.e., the phonological composite variable was described in Nakeva von Mentzer et al. (2013), and a reading composite variable and lexical expectation test were described in Nakeva von Mentzer et al. (2014b). Scores on key tests are presented in Table 1. The N400 procedure was identical across the two ERP sessions. Participants sat in front of a monitor at a distance of approximately 1 m. Each trial started with a fixation cross followed by a spoken word presented after 1 s. Word primes consisted of recorded spoken words (in Swedish) naming base-level common objects like foods, animals, clothes, body parts, vehicles, furniture, baby supplies, kitchen utensils and outdoor objects. Word primes were delivered at 75 dB (SPL). Picture targets were presented 2.3 s after word onset. After picture presentation participants indicated if the picture matched the word by pressing buttons on a response box corresponding to “yes” or “no.” This procedure was repeated for 120 trials. The procedure was introduced by a short training session including trials similar to those of the experimental paradigm, but without time limits for the response. When these trials were successfully completed, further trials included time limits for the response. Each stimulus pair consisted of a spoken prime followed by a picture target. The pairs were of three types, constituting the semantic conditions of the experiment: matches, where the target is a typical illustration of the prime word (e.g., “wolf” followed by a picture of a wolf), within-category mismatches, where the target is an illustration of another object than the prime, but from the same category or domain (e.g., “wolf” followed by a picture of a bear) or between-category mismatches where there is no apparent semantic link between prime and target (e.g., “wolf” followed by a picture of a car). There were 40 stimulus pairs in each condition, in total 120 pairs that were presented in mixed and random order. The pictures consisted of simple color drawings, depicting familiar objects in a cartoon-like or realistic manner. Pictures were presented on the screen against white background (width 12–18 cm and height 12–20 cm). Presentation and randomization of stimuli was handled by E-prime 2.0 software (Psychology 370 Software Tools Inc., 2012^1^; Pittsburgh, PA). Note that while the targets were pictures, the mismatch effects depend entirely on perceiving and deriving meaning from the spoken primes. A speech pathologist with experience working with DHH children prepared the words and pictures used as stimuli, the prime-target pairings, and recorded the spoken word stimuli. There was no quantitative matching of lexical, auditory or visual features of stimuli between conditions, as they were all very familiar base-level nouns and objects, chosen with intelligibility in mind. Stimulus pairs are described further in the Supplementary Materials.

The behavioral procedure was slightly revised after 10 of the included participants were tested (4 CI, 6 HA, 0 NC), after concerns that that use of the response box was confusing for some participants. Visual feedback was added in each trial, and a visual prompt for responses was omitted. All participants are included in the present analysis, but a complementary analysis excluding the first 10 participants is provided in the Supplementary Materials. This analysis reveals highly similar results and suggests that this subtle methodological change had no effect on outcomes.

### EEG recordings and processing

We recorded EEG at Department of Linguistics at Stockholm University, and at Humlab, Lund University using identical equipment from EGI (Electrical Geodesics Inc.), net amp 300 amplifier, electrode nets of the hydrocel 129 channel type (EOG channels were removed leaving 125 channels), using Cz as a reference channel and a ground channel positioned between CPz and Pz. In this system recordings are sampled at 20,000 Hz, low pass filtered online with a cut off at 4000 Hz and resampled to 250 Hz. The impedance of the channels was kept below 50 Ohm as recommended by the manufacturer. Hearing aids were refitted after the net was applied.

Recordings were filtered offline with a 1–40 Hz band pass FIR filter, resampled to 125 Hz and epoched. Only responses to picture targets were considered for the present study. Epochs with extreme amplitudes (exceeding ±500 μV) were rejected. Epoched data was subjected to preprocessing procedures in EP toolkit (Dien, 2010). Blink artifacts were removed with an automatic procedure, where independent component topographies are matched to a blink template. Movement artifacts were isolated using PCA and an amplitude criterion (i.e., principal components of single trial data with more than 200 μV amplitude change were removed). Channels with poor signal quality were identified globally (by means of correlation: correlation with neighboring channels should be above 0.4 but not perfect) and per epoch (amplitude differences within the epoch should be below 200 μV). Data from these channels were interpolated. A negligible number of artifacts could be attributed to CIs (Gilley et al., 2006; Debener et al., 2008; Viola et al., 2012) and did not demand special treatment. In total, 6% of all trials were rejected, and each subject retained on average 220 trials (*SD* = 28) of 240.

## Results

**Figure 1:**
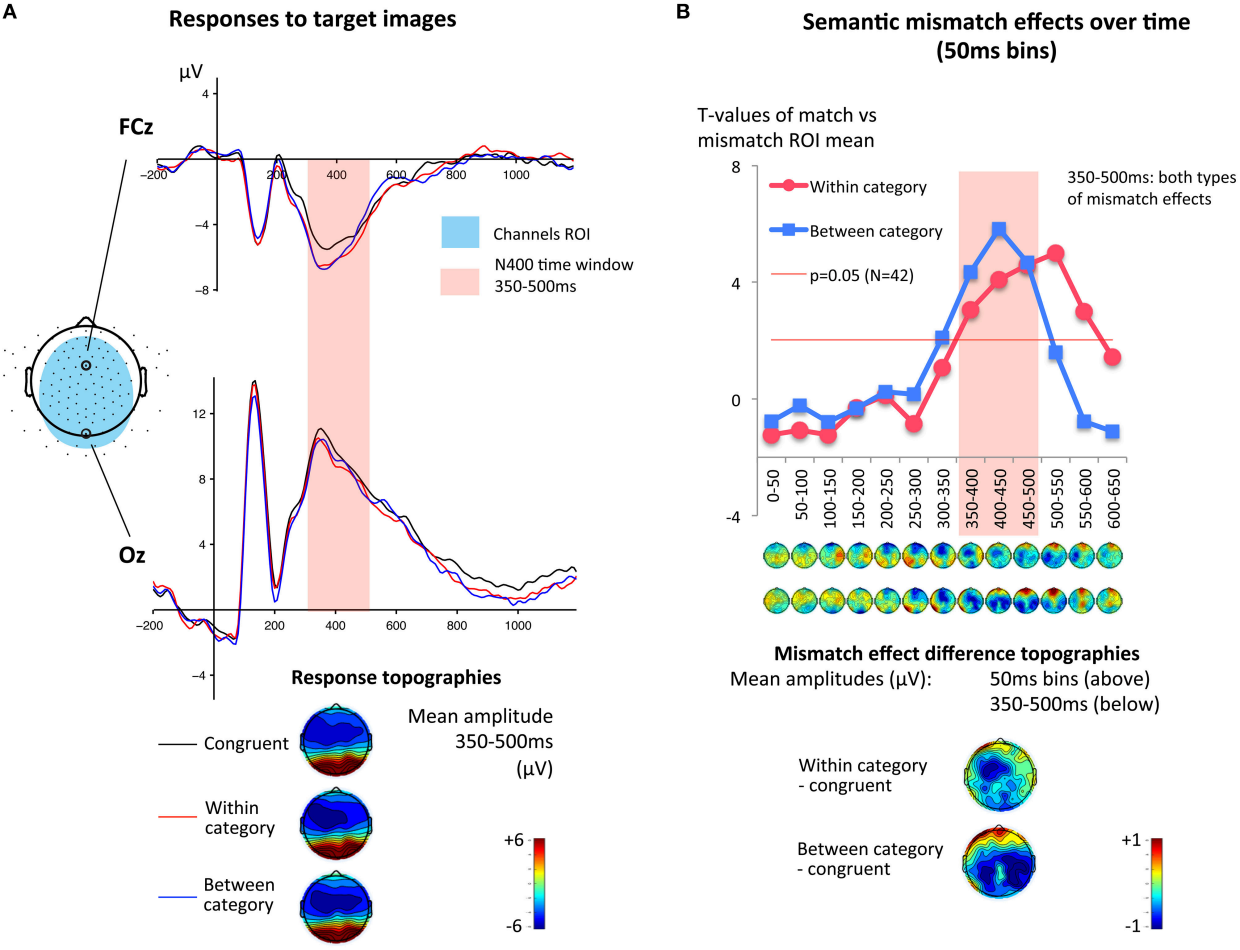
**(A)** Overview of ERP responses: average responses at FCz and Oz, and topographic maps for the N400 time window 350–500 ms. Averages include all participants, collapsed across pre- and post-intervention. **(B)**. Within- and between-category mismatch effects (mismatches compared to matches) over time. *T*-tests based on amplitude averages in 50 ms time windows in the Region of Interest (ROI). Both effects were found in the time window 350–500 ms, which was used for the main analysis. Topographic maps show the mismatch effect amplitude differences, in 50 ms time windows and in the critical 350–500 ms interval.

First, we established the presence of semantic mismatch effects in the EEG data. Thus, data (collapsed across groups and intervention conditions) were visually inspected, and showed a large negative fronto-central peak in responses to pictures in all semantic conditions. Difference waves, produced by subtracting responses to matching pictures from responses to mismatching pictures, showed a broad negative deflection and polarity shift at lateral sites. A broad electrode window was used to capture these effects (all electrodes except edges and lateral sites, see Figure 1A). Only one electrode region was used, in order to reduce number of factors in the ANOVA (Luck, 2014). To obtain information about the time course of this semantic differential, average amplitudes of these electrodes were tested for semantic mismatch effects in series of *T*-tests, using 50 ms bins, from 0 to 650 ms after stimulus onset. Between 350 and 500 ms, both mismatch types differed significantly from congruent responses (see Table 2B), constituting an overall semantic mismatch effect in line with typical N400 descriptions (Picton et al., 2000; Kutas and Federmeier, 2011). *T*-values over time bins are presented in Figure 1B with separate lines for within-category and between-category mismatches, each compared to the congruent responses. While significance levels presented in Table 2B are uncorrected, the N400 difference results remain significant at the 0.05 level (one-tailed) also with Bonferroni-correction (corrected *p*-value 0.05/26 = 0.002 and critical *t*-value at 3.05).

**Table 2:**
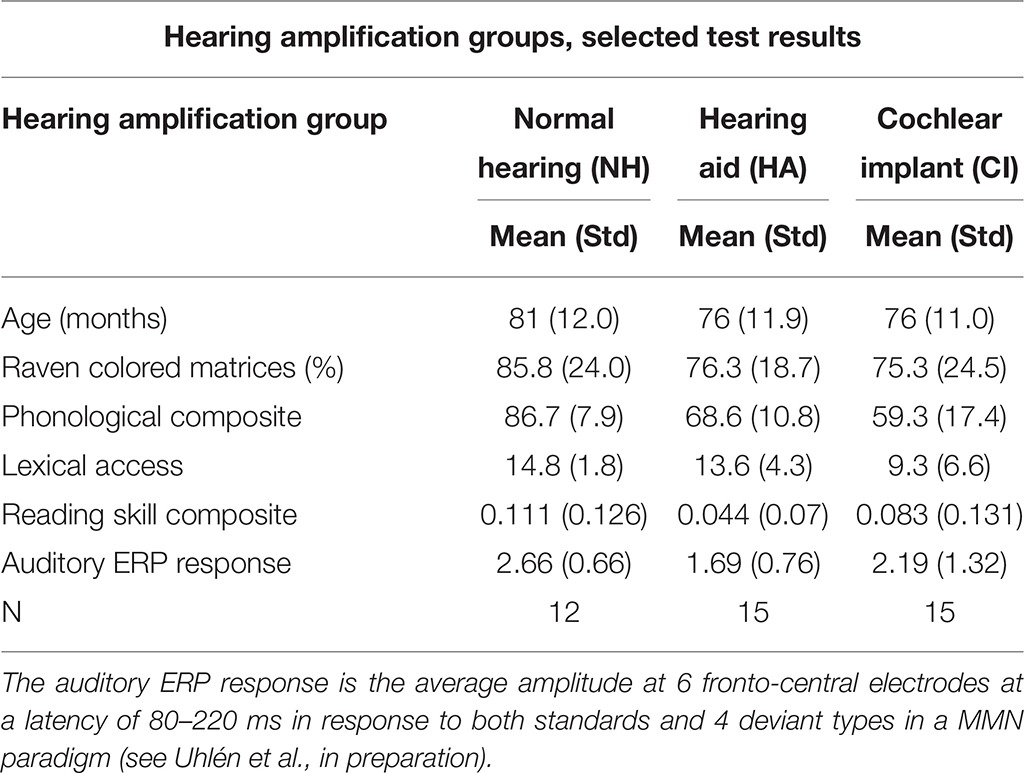
**(A) Main effects and interactions of semantic conditions, training and group. Significant effects, and the nonsignificant effect of training is included. (B) Mismatch effects were assessed in 50 ms time windows across all participants, to establish the time-window of the effects (dotted square). Group-specific tests explored the semantic condition × group interaction over time. (C) Explorative correlation of peak mismatch effects and language test variables**.

^*^p < 0.05, ^**^p < 0.01, ^***^p < 0.001 uncorrected P-values.

**Figure 2:**
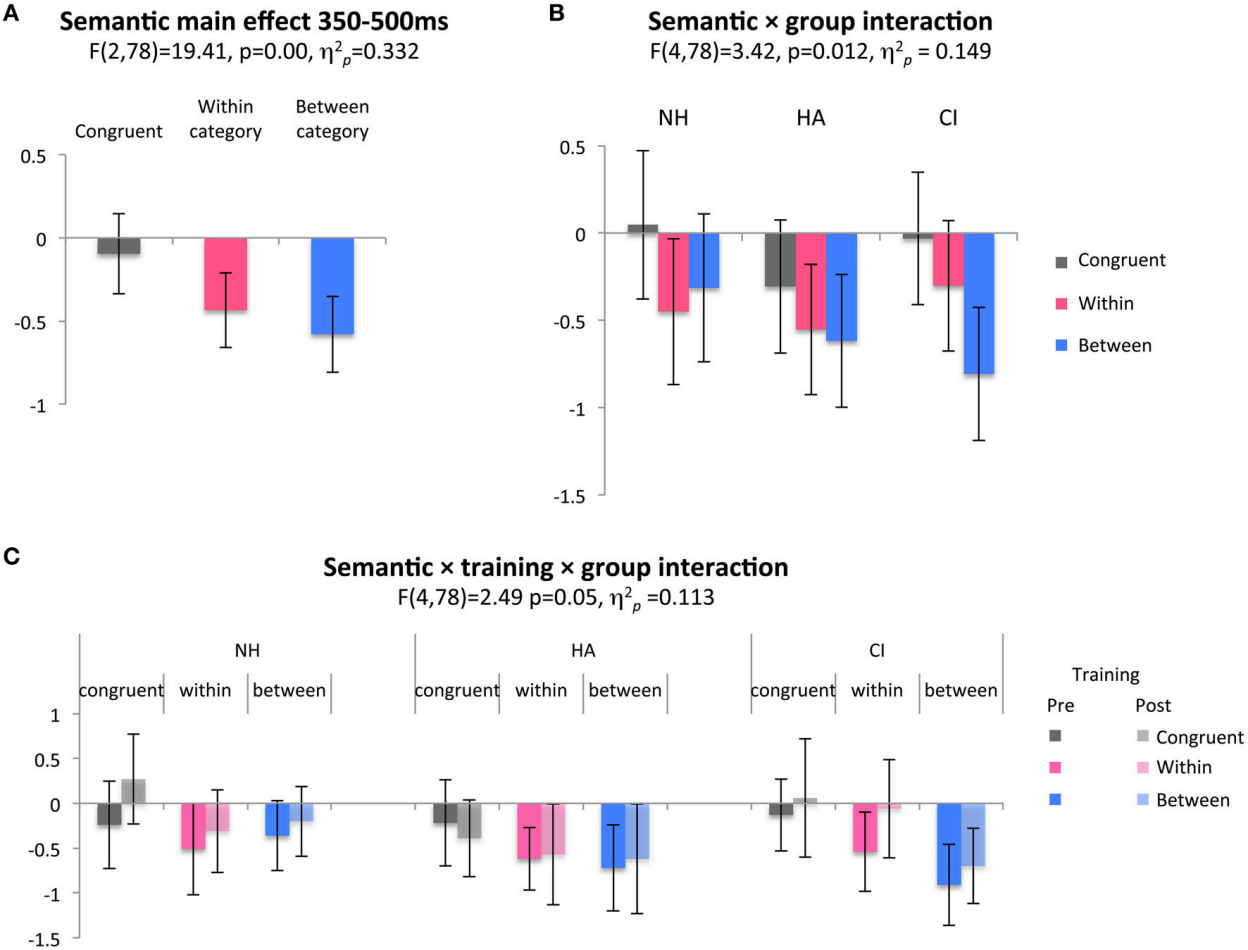
**(A)** Main ERP effect of semantic condition revealing less negative responses to congruent images, and more negative responses in mismatches of both types. **(B)** Group interaction showing similar responses to both mismatch types in controls and children with HA, while children with CI show a distinct response. **(C)** The three-way interaction shows differences in the group pattern before and after training. However, mismatch effects for DHH children are not emphasized after training, so no positive effect of intervention can be inferred from this interaction.

The 350–500 ms time window was used for the main analyses to assess group differences in the N400 response in relation to semantic difficulties among DHH children. To this end, three factors were analyzed in a repeated-measures ANOVA: semantic incongruence (within-category, between-category, and congruent), intervention (before and after) and hearing amplification group (CI, HA, and NH). There was a main effect of semantic condition (Figure 2A, Table 2A), again confirming an overall semantic N400 effect among the participants, where the congruent condition differed from both mismatch types. There was also an interaction between semantic condition and group: Whereas between-category vs. congruent trials displayed the largest mismatch effect for the CI group, within- and between-category mismatch types were similar for the NH and HA groups. The HA group had less pronounced mismatch effects overall (Figure 2B). There was no meaningful main effect of intervention, or of the intervention factor interacting with semantic condition. A three-way interaction between group, semantic condition and intervention reached significance (Figure 2C). In the pre-training session, responses were similar among groups except for between-category responses (where the response was largest for the CI group and smallest for NH children). In the post-training session, groups were more different, with both mismatch responses larger than before for NH, almost no differences among children with HA and essentially the same response to congruent and within-category incongruent for CI-users (still with a large between effect). This pattern does not fit predictions of improved semantic processing for DHH children due to the intervention. Rather, it indicates that group differences in the first session were somewhat enhanced in the second. In sum, the analysis confirmed typical N400 incongruence effects, and show differences in semantic processing among groups. Smaller mismatch effects overall for HA-users, and little within-category mismatch effect for CI-users, despite a large between-category effect, is broadly in line with predictions of less semantic sensitivity among DHH children. However, the result that the between-category mismatch response was larger for children with CI than for NH was unexpected and might suggest differences in processing mode rather than a lack of semantic competence in the NH group.

**Figure 3:**
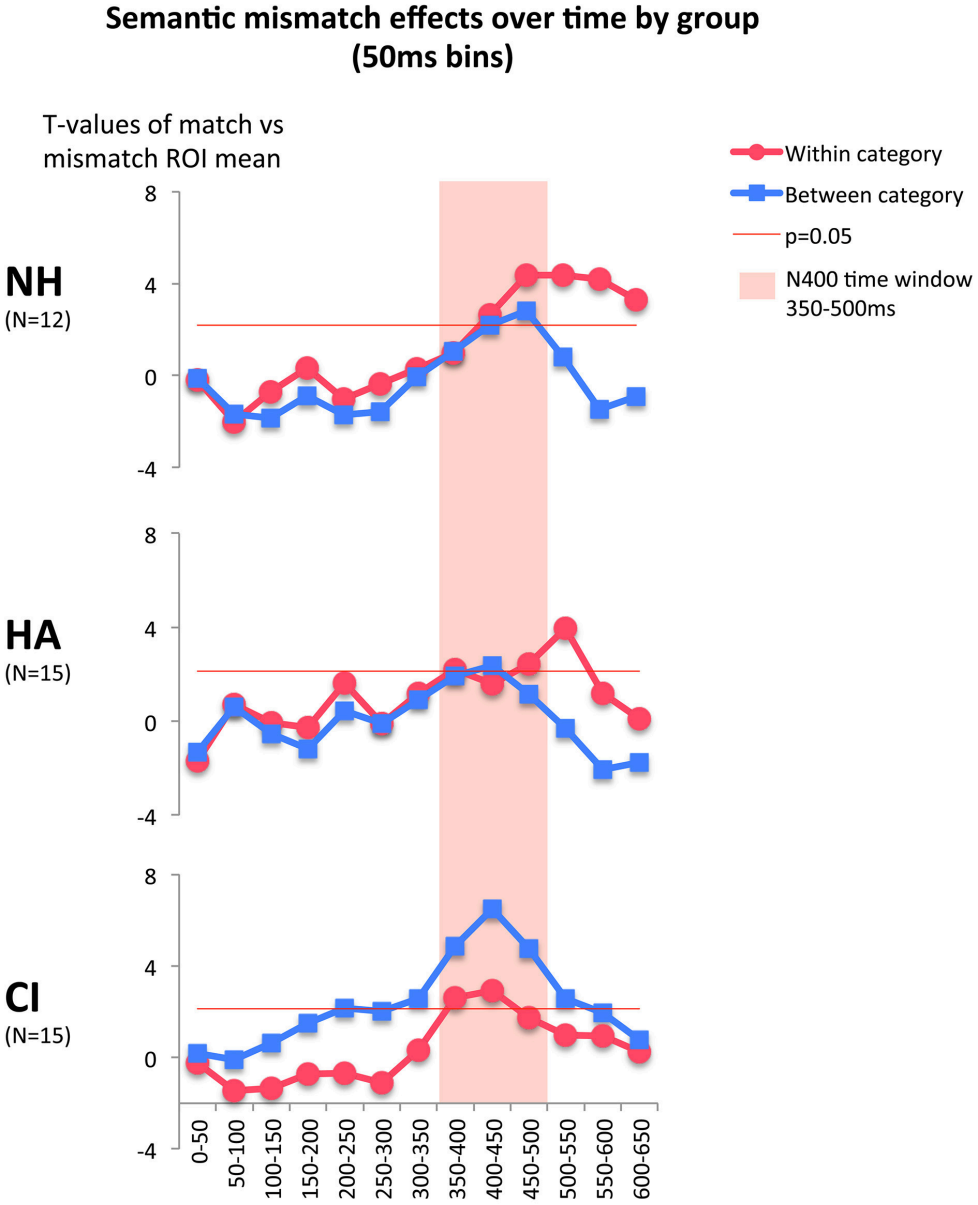
**Within- and between-category mismatch effects (mismatching trials compared to congruent trials) over time**. *T*-tests based on ERP amplitude averages in 50 ms time windows in the Region of Interest (ROI). Positive threshold for *p* = 0.05 is shown for *N* = 12 (NH group) or *N* = 15 (HA and CI groups). A strong between-category mismatch effect is seen in children with CI, and a late within-category effect in children with NH and HA. Only the positive threshold is plotted, but one point with a negative *T*-value does reach the negative threshold for significance (the second red dot, 50–100 ms for NH children).

In order to understand the time courses of semantic processing, we explored group differences further by reapplying the serial *T*-test analysis to each group separately (Figure 3). In the original series of *T*-tests, presented in Figure 1B, a difference was present in the time courses of within- and between-category mismatch effects. The between-category mismatch effect peaked at 400–450 ms whereas the within-category mismatch peaked at 500–550 ms, 100 ms later. As showed in Figure 3, mismatch effects in the group of children with CI peaked at 350–500 ms (in the time window of our main analysis) with much larger effects for between-category mismatches. In contrast, children with NH and HA showed larger effects for within-category mismatches, peaking after 500 ms. The effects showed small to moderate positive correlations with behavioral tests of semantic and phonological skills. The within-category mismatch effect at 500–550 ms was negatively correlated with test scores of lexical expectations and phonological skills. The between-category effect at 400–450 ms showed smaller positive correlation to the same variables. The within-category correlations, but not the between-category correlations, were statistically significant (see Table 2C). Two other potentially interesting variables, participant age and reading composite score, were tested for significant correlations, but none were found. The exploratory analysis highlighted the distinct cortical response pattern of the CI group, seen in the main analysis, and showed that response patterns among children with NH and HA were similar, with an extended within-category effect that was associated with lexical processing skills. These exploratory *T*-tests and correlations were presented without correction for multiple comparisons.

## Discussion

Semantic processing in DHH children has been largely unexplored at the neural level. In our word-picture matching design, both DHH children and NH controls showed large negative deflections for mismatching target pictures, consistent with typical N400 effects. This group study of N400 responses in children with CI, support the observations in a previous case report (Key et al., 2010). In our results, based on N400 responses to visual stimuli, we did not observe the prolonged N400 latencies previously reported among adult CI users in response to speech (Hahne et al., 2012; Henkin et al., 2015; Finke et al., 2016), perhaps due to stimulus modality differences across studies. At a more detailed level, we observed differences between controls, children with HA and children with CI. Children with HA had nominally smaller mismatch effects than those of other groups, especially post-intervention (see Figure 2C). It is possible that the group with HA did not hear the primes as well as other participants, because they had smaller ERP responses to tones also in a subsequent auditory mismatch negativity paradigm (see Table 1 and Uhlén et al., in preparation). The fact that ERP mismatch effects declined between recording sessions might, however, be more consistent with a diminishing motivation specifically for this group, although this was not apparent during interaction with the children. Future studies will ultimately show if this result is reproducible or, as we suspect, was a spurious finding.

Unexpectedly, children with CI had a larger between-category mismatch effect than the other groups. In some settings, a large mismatch effect would indicate better semantic discrimination, but given that the participants with CI did not perform well on a lexical prediction test (Nakeva von Mentzer et al., 2014b) this is unlikely. In contrast, children with preserved hearing and semantic ability had smaller ERP mismatch effects, with similar responses to both mismatch types. The exploratory *T*-tests revealed how the between-category effect had an early maximum and was largest for children with CI, while the within-category effect was largest among children with NH and HA and had a later maximum for these groups. The amplitude of the latter effect was correlated with better phonological and lexical skills (see Table 2C). The differences in timing and magnitude of N400 effects might indicate that children with CI engage in the task with different processing modes or strategies than children with NH and HA.

Prior work has found that lack of predictive processing might affect ERPs such that semantic within-category and between-category effects become more alike (Federmeier and Kutas, 1999; Franklin et al., 2007; Wlotko et al., 2010; Kiang et al., 2013). Effects of semantic relatedness on ERPs are typically smaller or absent when semantic processing results from passive, bottom-up processing, when motivation is lower (Kiang et al., 2013), at older age (Wlotko et al., 2010), or when the stimulated visual field favors processing outside of the language-dominant left-hemisphere (Federmeier and Kutas, 1999). Although our results are not conclusive, we speculate that children with CI might rely on more predictive processing than controls when performing this task. Predictive processing is a successful strategy used by this group to solve auditory tasks (Lyxell et al., 2009; Nakeva von Mentzer, 2014). Furthermore, the task design included only one-third matching trials, and one-third of trials were semantically challenging, within-category mismatches. It is possible that controls soon realize that primes do not accurately predict targets except in a minority of cases, and switch to a more passive, bottom-up mode. As the task is much more challenging for children with CI, they might be less likely to identify the low proportion of matches, and more likely to stay in a predictive mode even if it is more effortful. Children with CI have less structured semantic relations between word meanings (Kenett et al., 2013), which means that within-category mismatches will be mistaken for matches to some extent, and possess features that are overlapping with the predicted match, leading to a reduction of N400 amplitude. In sum, one possible explanation of our observed group differences is that children with CI rely on a predictive processing mode that reflects motivated effort. In everyday communication this might be an adaptive strategy, but in the present experiment it is not. Controls, in contrast, might use a more passive bottom-up processing mode that is more adaptive in this context.

The differentiated mismatch effects among CI children might be interpreted as a reflection of lower semantic precision, in line with prior work (Kenett et al., 2013). However, we find this interpretation unsatisfactory, because a lack of mismatch differentiation could also reasonably be interpreted as a lack of semantic precision. A third possible explanation for the absence of relatedness effects among NH children is that the mismatch response to between-category targets are influenced by a P3b component (Polich, 2007) that overlaps with the N400. As our results were unexpected, we encourage future studies to investigate whether predictive processing, an overlapping P3b response, or other interpretations could account for the deviating between-category mismatch effect in the N400 responses of individuals with CI.

In conclusion, our findings indicate that the ERP-responses of semantic processing in DHH children share similarities with those of controls. However, there are differences that seem to reflect different responses to task demands. The relatively large and differentiated N400 mismatch effects among children with CI could reflect predictive, top-down semantic processing. If we accept this interpretation, our results, together with the lack of positive effects of the phonics training intervention on the N400, emphasize the role of top-down semantic processing, and would highlight strategies such as perspective guiding in teaching reading comprehension to DHH individuals (Luckner and Handley, 2008). Further studies could use paradigms similar to ours to link top-down semantic processing closer to specific patterns of brain responses and behavioral results. Ways of supporting an adaptive use of such processing strategy should be investigated, perhaps by investigating the role of feedback on performance and ERP responses.

## Author contributions

PK adapted the experiment, collected and analyzed ERP data and wrote the manuscript. JO supervised writing and analysis. ML, BS, BL, IU, MO conceived and designed the experiment. MO, EE, CN collected and managed the data. CN, PK, and BL analyzed behavioral data. CN, ML, BS, IU, EE read and commented on manuscript.

### Conflict of interest statement

The authors declare that the research was conducted in the absence of any commercial or financial relationships that could be construed as a potential conflict of interest.

## Footnotes

^1^Psychology Software Tools Inc. (2012). *Retrieved from* http://www.pstnet.com.
