# Supplementary material for: Semantic Processing in Deaf and Hard-of-Hearing Children: Large N400 Mismatch Effects in Brain Responses, Despite Poor Semantic Ability
Source: Front Psychol. 2016 Aug 10;7:1146. doi: 10.3389/fpsyg.2016.01146 (PMC4978721; doi:10.3389/fpsyg.2016.01146)
Supplement: Supplementary file 1 [file Presentation1.pptx]

## Slide 1
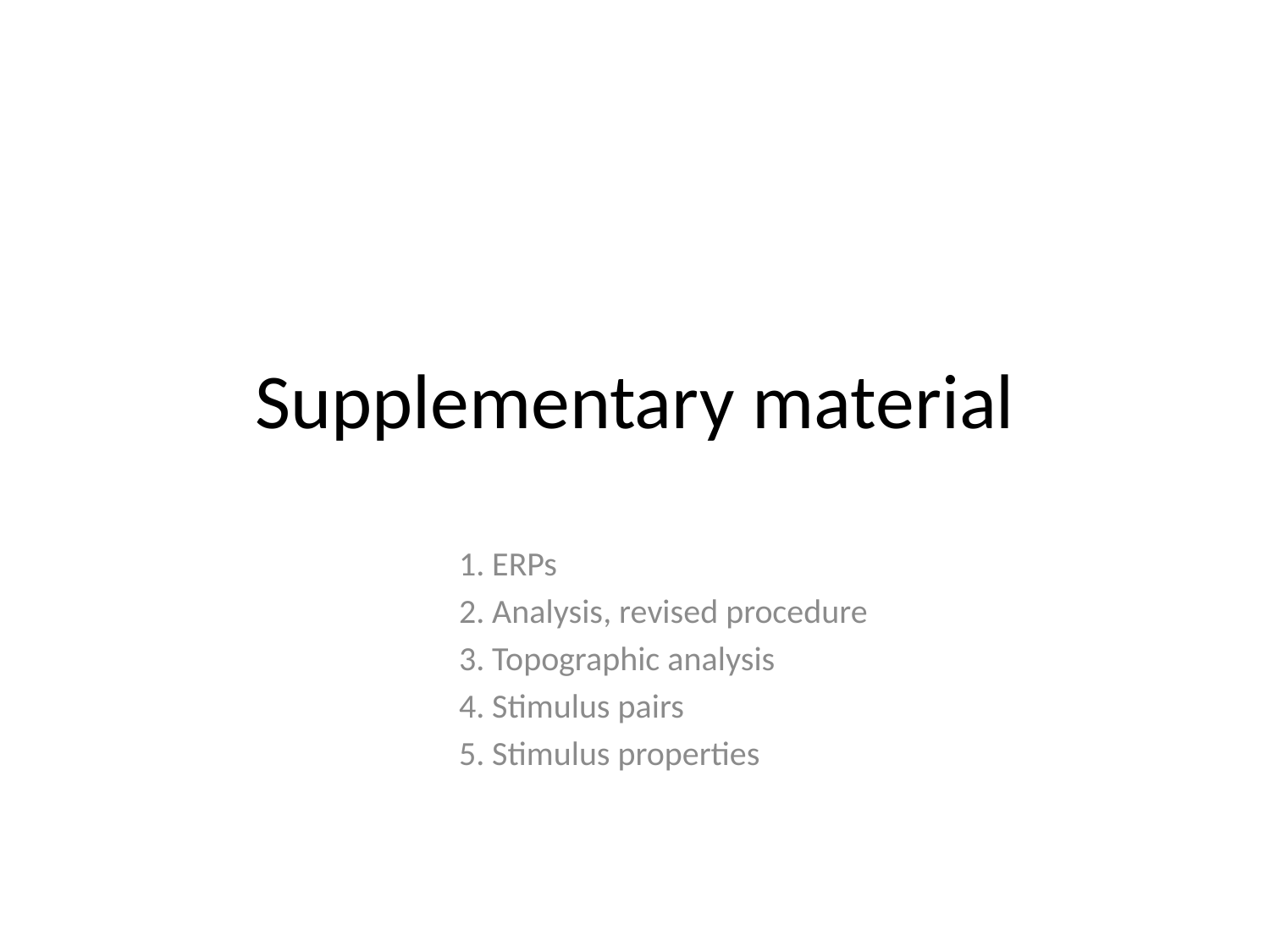

# Supplementary material
1. ERPs
2. Analysis, revised procedure
3. Topographic analysis
4. Stimulus pairs
5. Stimulus properties

## Slide 2
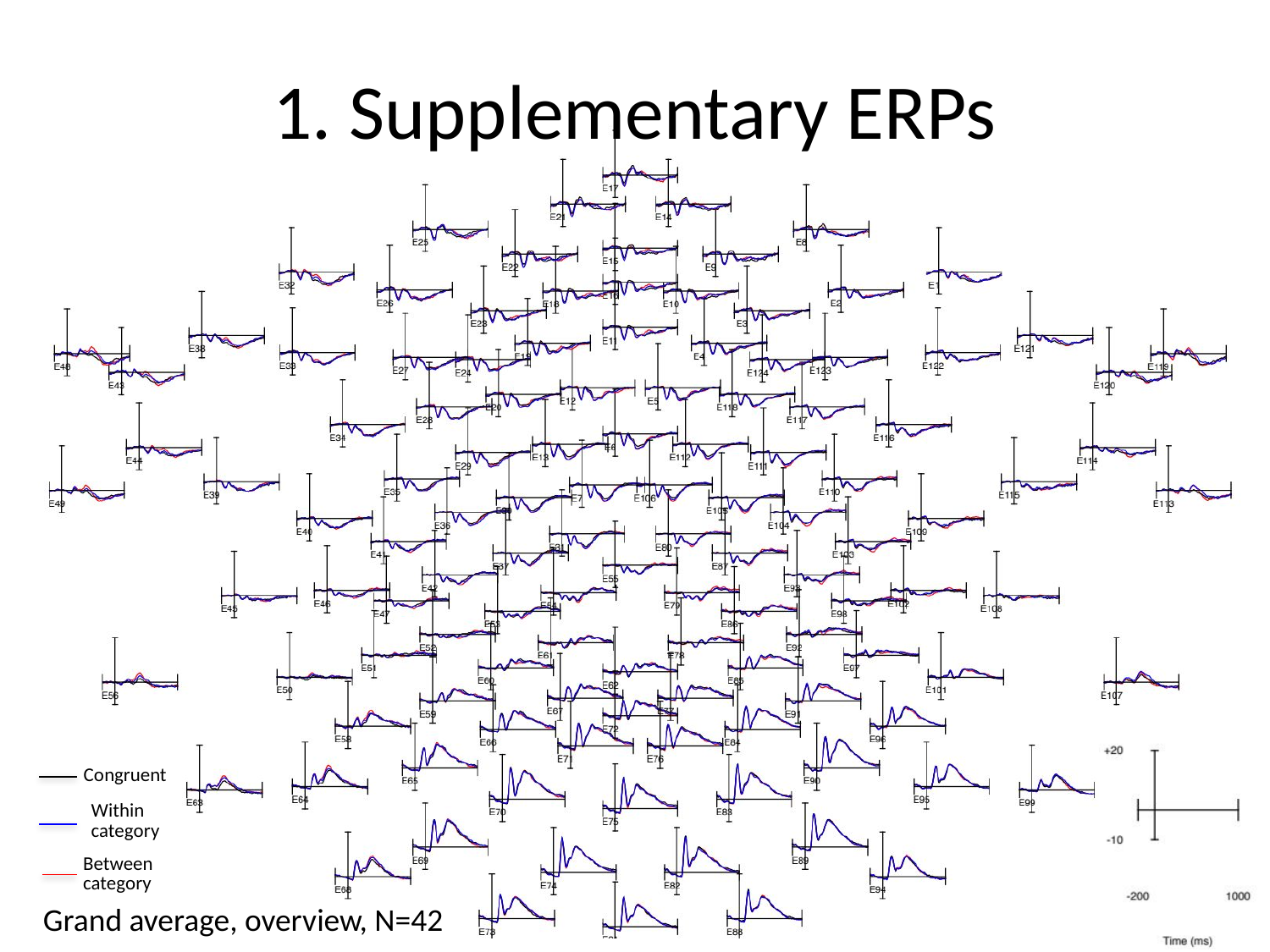

# 1. Supplementary ERPs
Congruent
Within category
Between category
Grand average, overview, N=42

## Slide 3
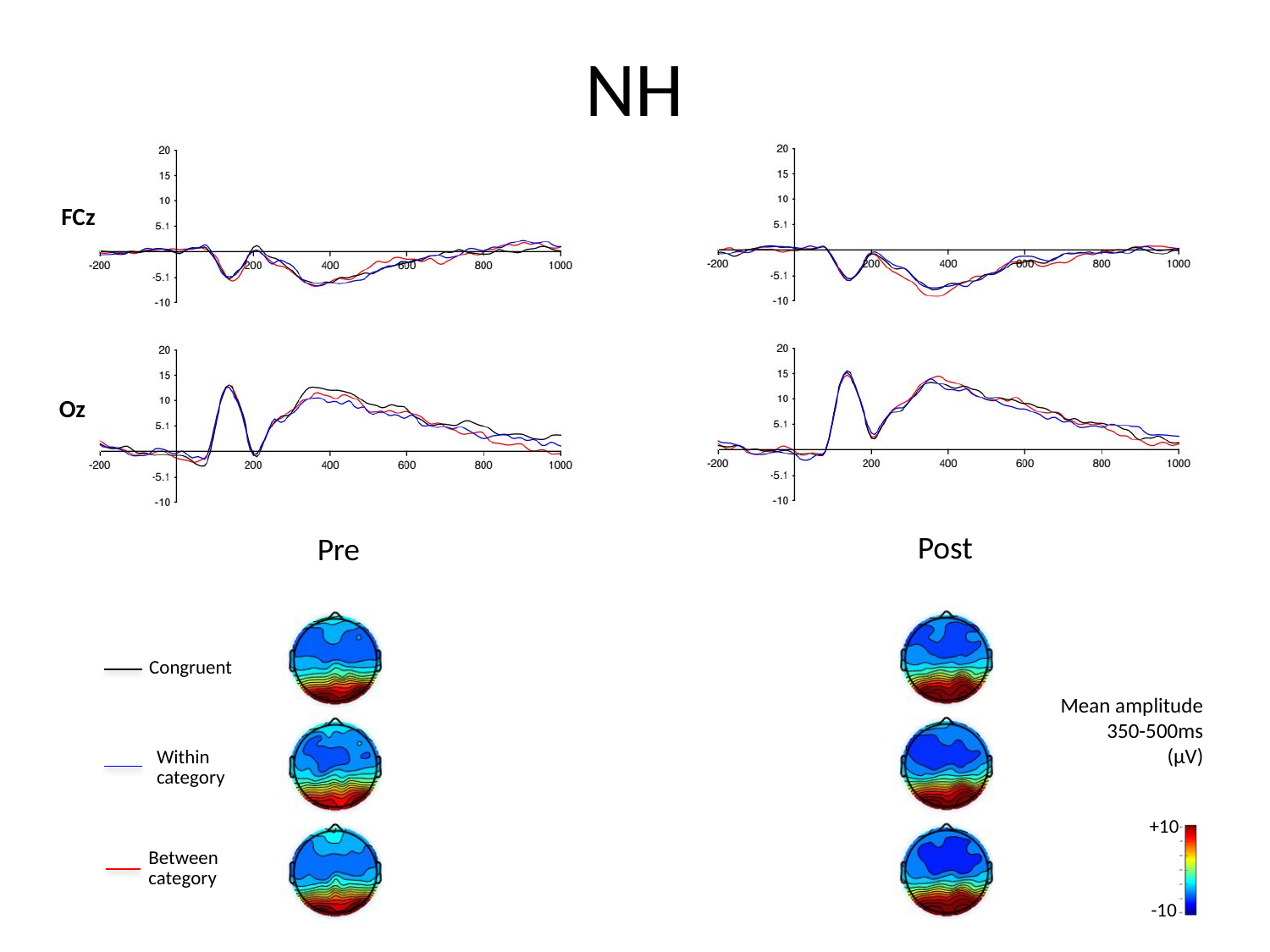

# NH
FCz
Oz
Post
Pre
Congruent
Mean amplitude
350-500ms
(μV)
Within category
+10
Between category
-10

## Slide 4
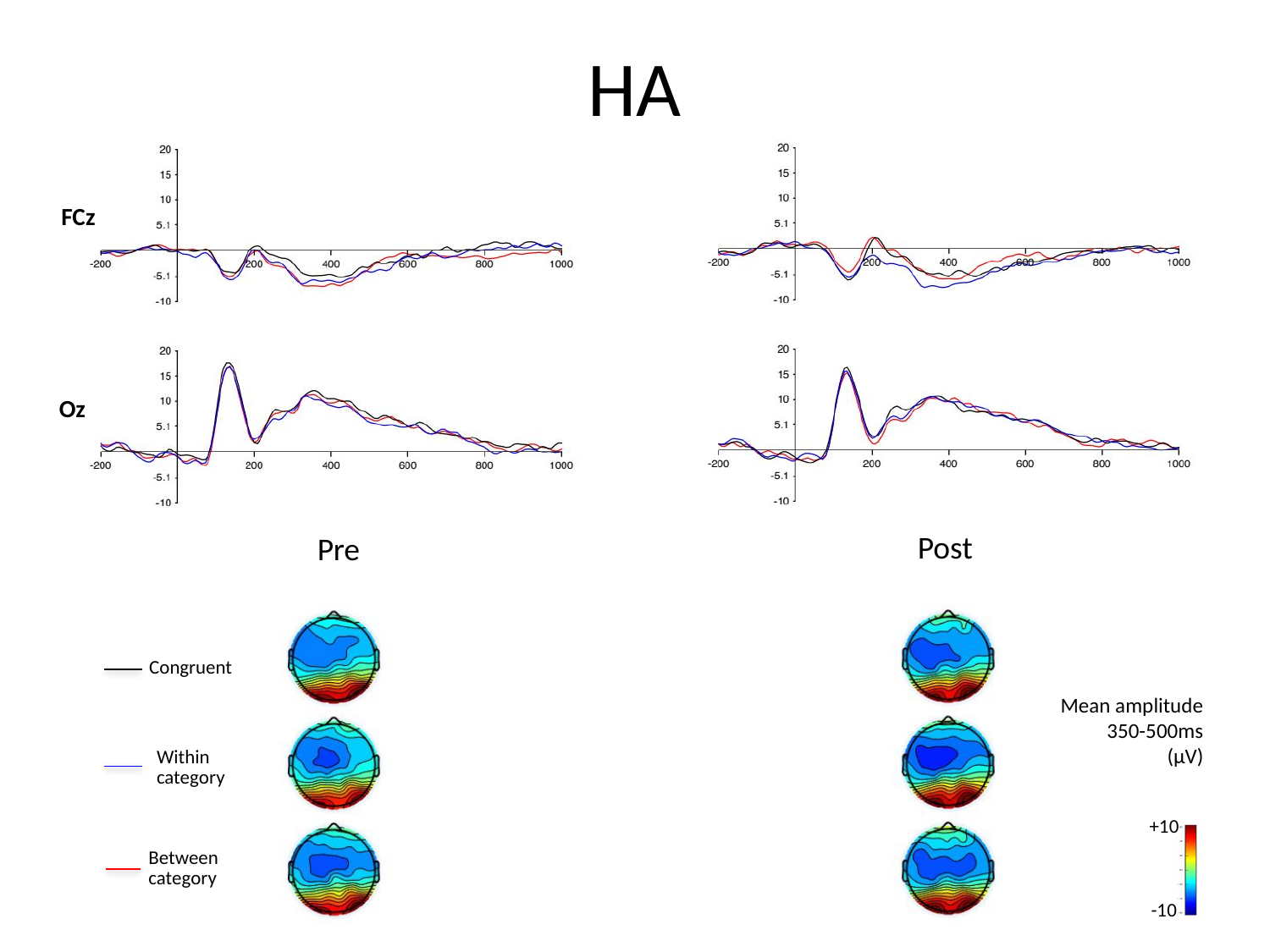

# HA
FCz
Oz
Post
Pre
Congruent
Mean amplitude
350-500ms
(μV)
Within category
+10
Between category
-10

## Slide 5
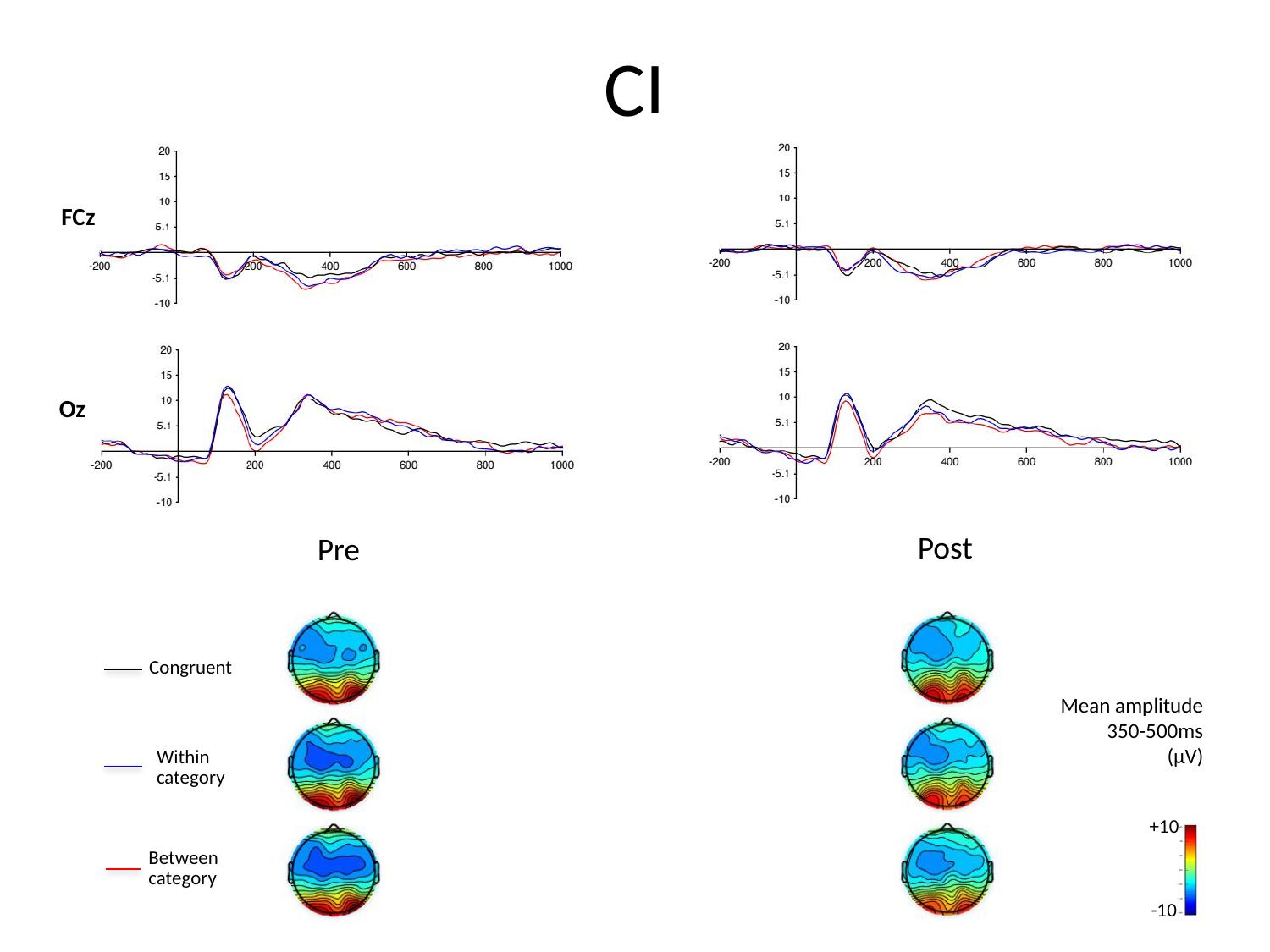

# CI
FCz
Oz
Post
Pre
Congruent
Mean amplitude
350-500ms
(μV)
Within category
+10
Between category
-10

## Slide 6
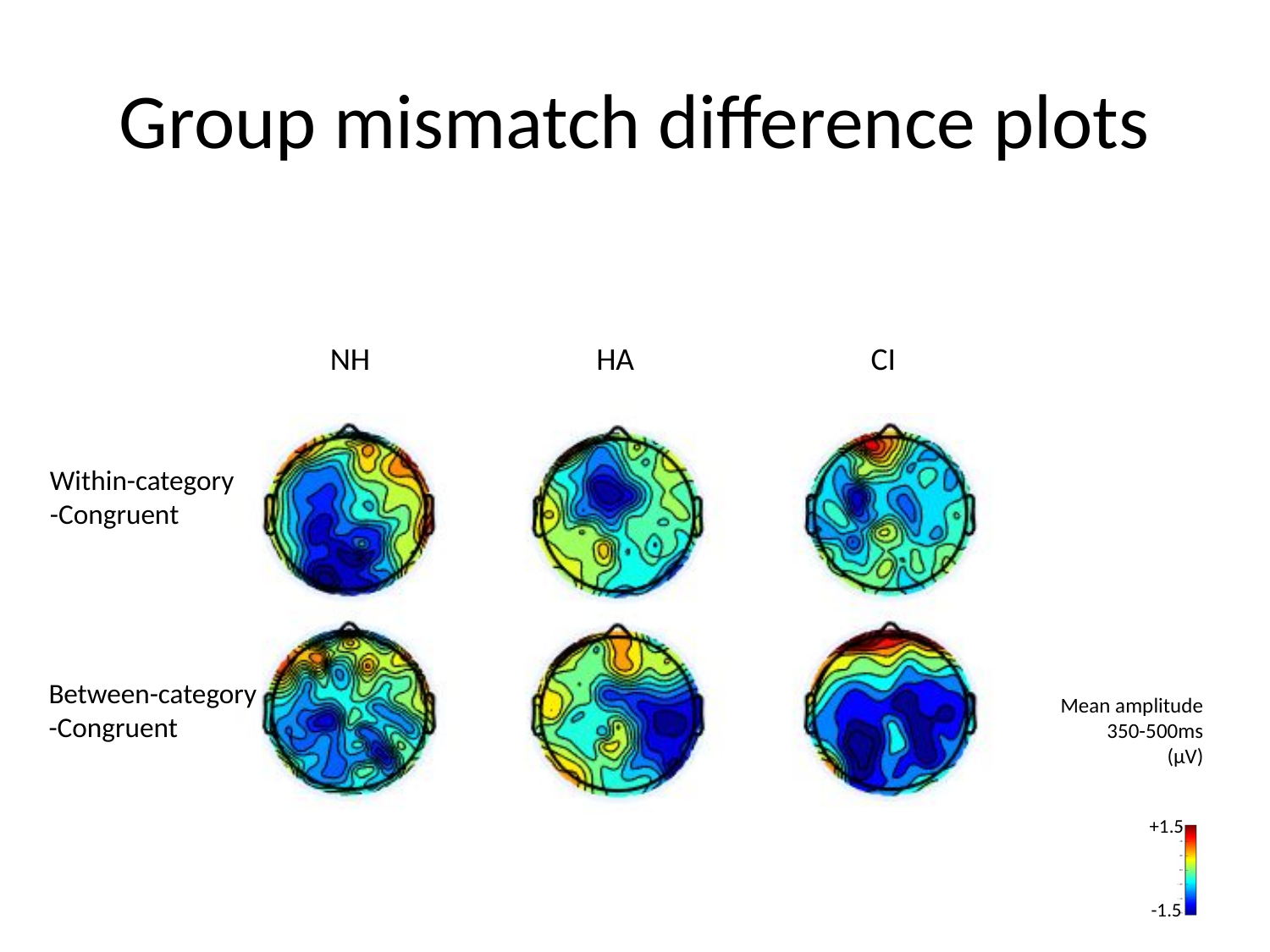

# Group mismatch difference plots
NH
HA
CI
Within-category
-Congruent
Mean amplitude
350-500ms
(μV)
Between-category
-Congruent
+1.5
-1.5

## Slide 7
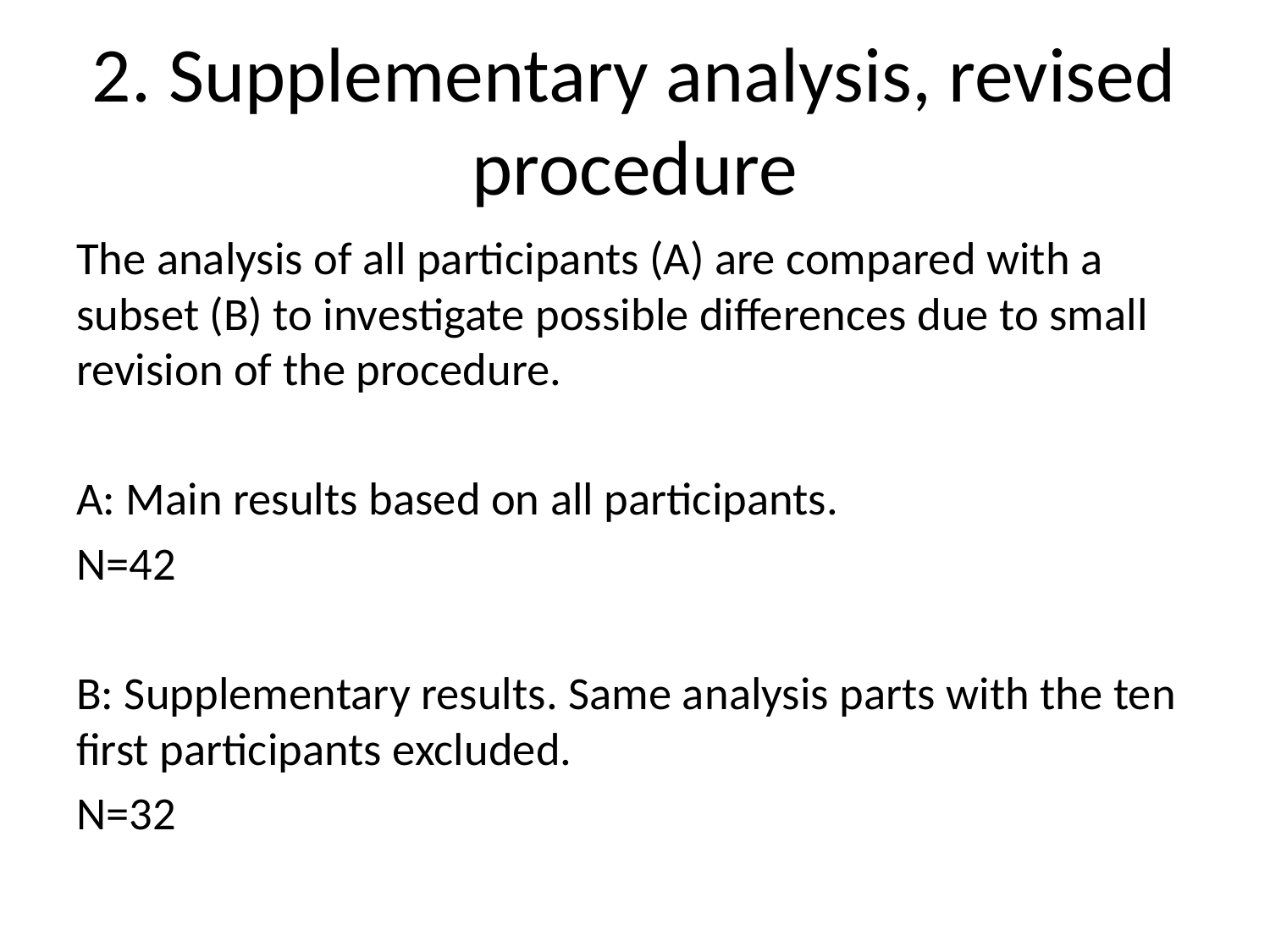

# 2. Supplementary analysis, revised procedure
The analysis of all participants (A) are compared with a subset (B) to investigate possible differences due to small revision of the procedure.
A: Main results based on all participants.
N=42
B: Supplementary results. Same analysis parts with the ten first participants excluded.
N=32

## Slide 8
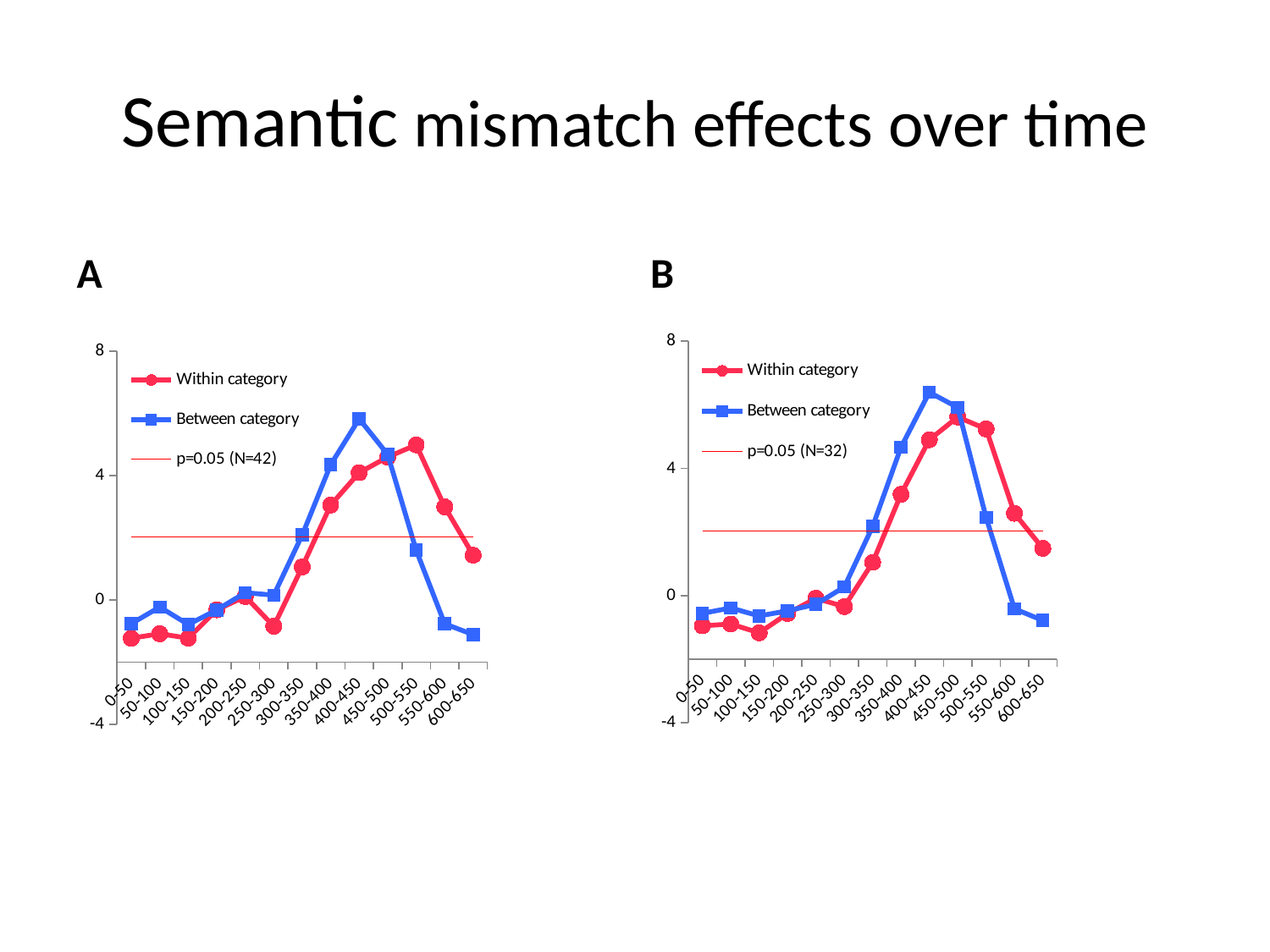

# Semantic mismatch effects over time
A
B
### Chart
| Category | Within category | Between category | p=0.05 (N=32) |
|---|---|---|---|
| 0-50 | -0.951 | -0.558 | 2.04 |
| 50-100 | -0.894 | -0.388 | 2.04 |
| 100-150 | -1.171 | -0.641 | 2.04 |
| 150-200 | -0.565 | -0.478 | 2.04 |
| 200-250 | -0.084 | -0.273 | 2.04 |
| 250-300 | -0.347 | 0.27 | 2.04 |
| 300-350 | 1.044 | 2.178 | 2.04 |
| 350-400 | 3.188 | 4.665999999999995 | 2.04 |
| 400-450 | 4.903 | 6.395 | 2.04 |
| 450-500 | 5.616 | 5.917 | 2.04 |
| 500-550 | 5.243 | 2.461 | 2.04 |
| 550-600 | 2.589 | -0.414 | 2.04 |
| 600-650 | 1.487 | -0.782 | 2.04 |
### Chart
| Category | Within category | Between category | p=0.05 (N=42) |
|---|---|---|---|
| 0-50 | -1.23 | -0.761 | 2.0181 |
| 50-100 | -1.084 | -0.225 | 2.0181 |
| 100-150 | -1.228 | -0.786 | 2.0181 |
| 150-200 | -0.315 | -0.32 | 2.0181 |
| 200-250 | 0.111 | 0.235 | 2.0181 |
| 250-300 | -0.843 | 0.156 | 2.0181 |
| 300-350 | 1.067 | 2.092 | 2.0181 |
| 350-400 | 3.055 | 4.353 | 2.0181 |
| 400-450 | 4.095 | 5.824999999999995 | 2.0181 |
| 450-500 | 4.597 | 4.68 | 2.0181 |
| 500-550 | 4.989 | 1.6 | 2.0181 |
| 550-600 | 2.997 | -0.766 | 2.0181 |
| 600-650 | 1.442 | -1.113 | 2.0181 |

## Slide 9
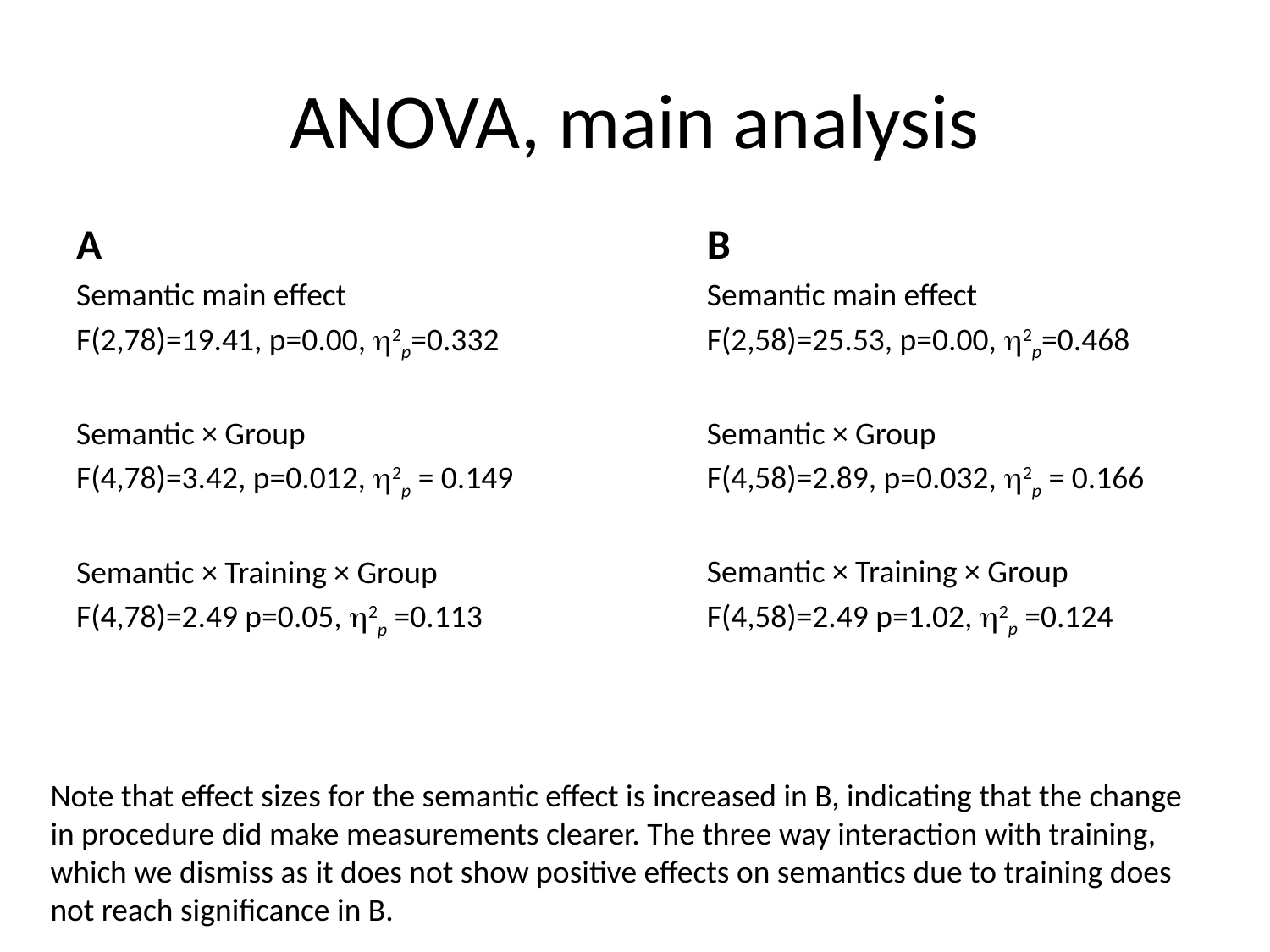

# ANOVA, main analysis
A
Semantic main effect
F(2,78)=19.41, p=0.00, 2p=0.332
Semantic × Group
F(4,78)=3.42, p=0.012, 2p = 0.149
Semantic × Training × Group
F(4,78)=2.49 p=0.05, 2p =0.113
B
Semantic main effect
F(2,58)=25.53, p=0.00, 2p=0.468
Semantic × Group
F(4,58)=2.89, p=0.032, 2p = 0.166
Semantic × Training × Group
F(4,58)=2.49 p=1.02, 2p =0.124
Note that effect sizes for the semantic effect is increased in B, indicating that the change in procedure did make measurements clearer. The three way interaction with training, which we dismiss as it does not show positive effects on semantics due to training does not reach significance in B.

## Slide 10
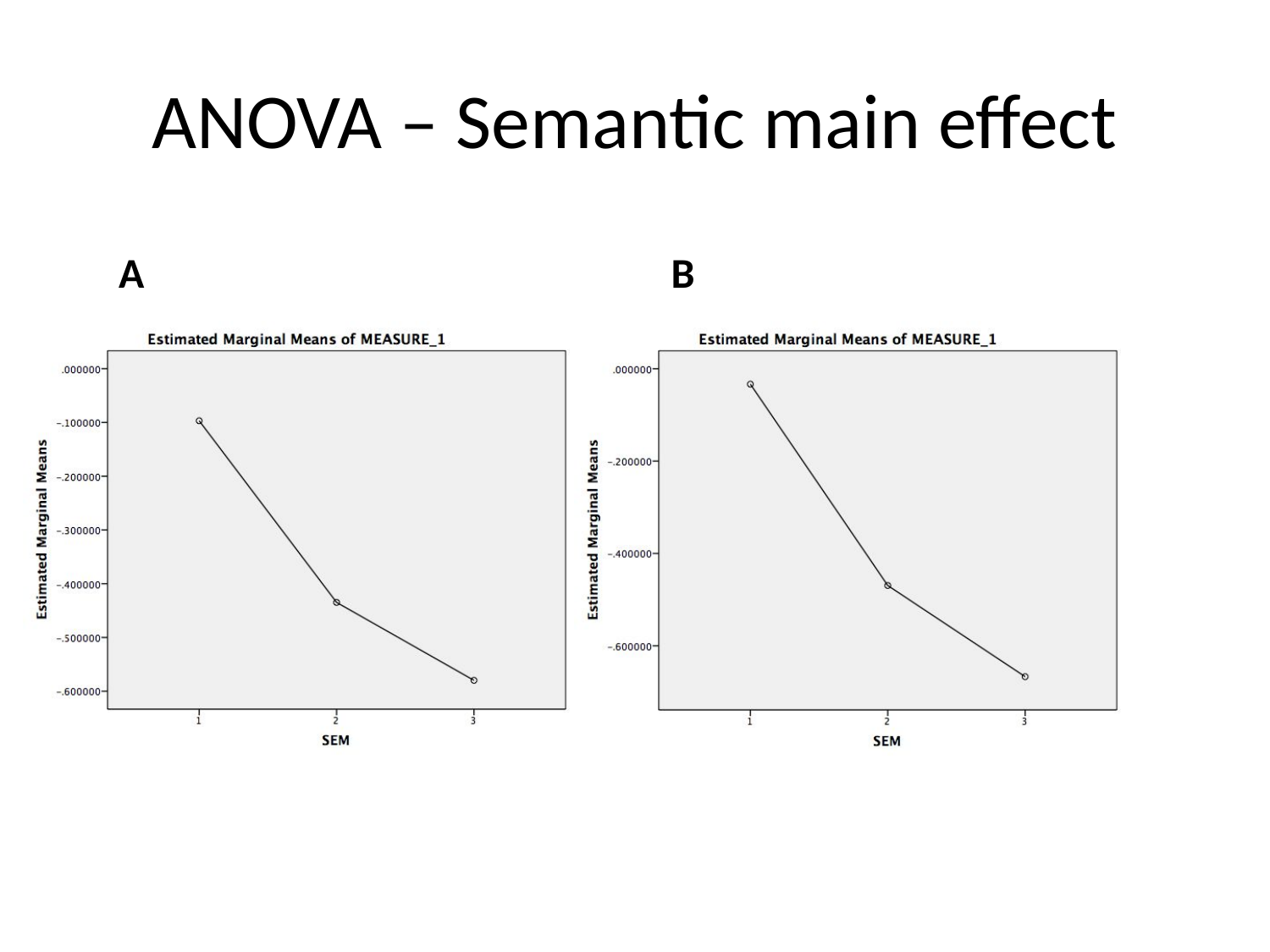

# ANOVA – Semantic main effect
A
B

## Slide 11
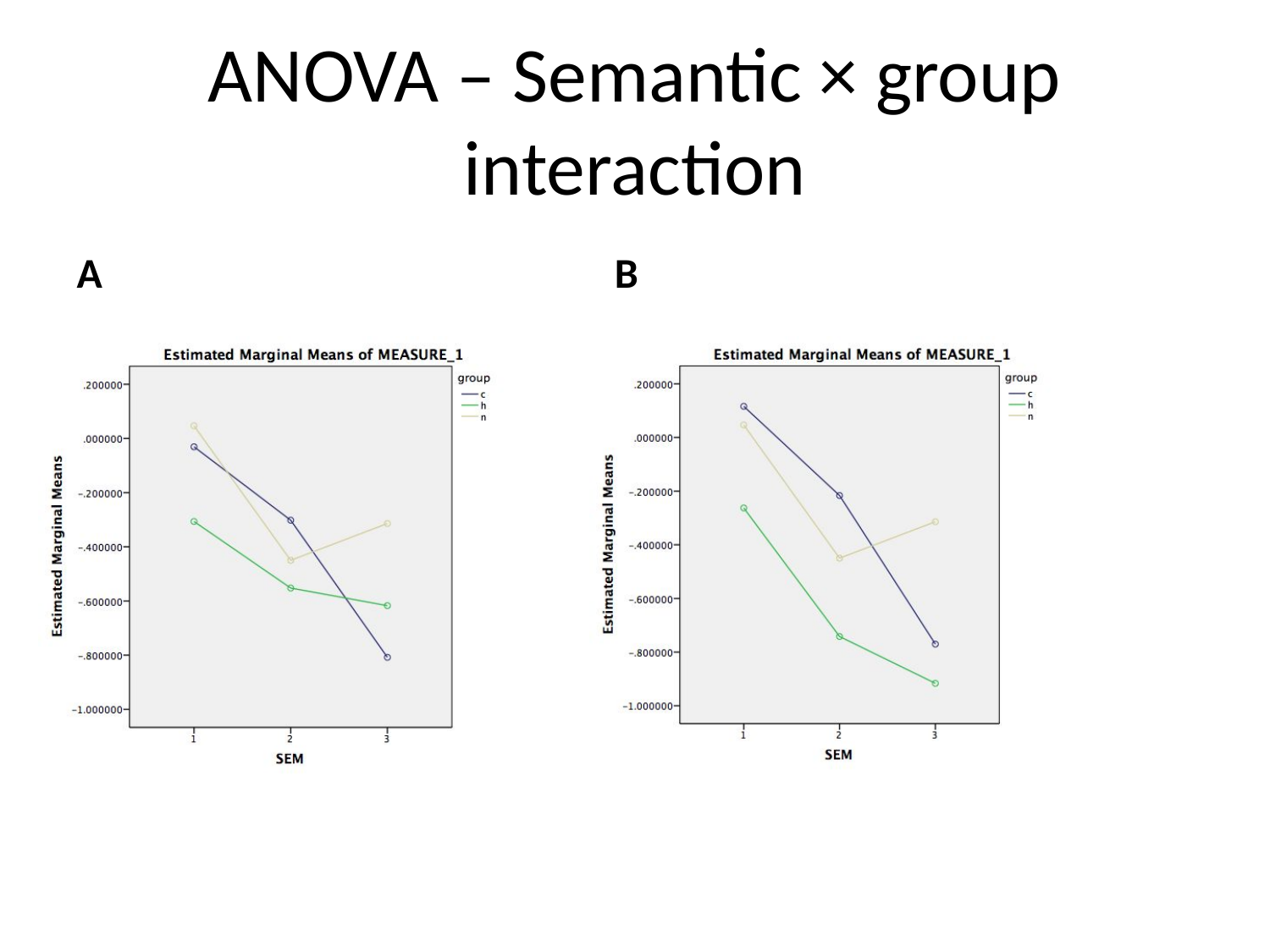

# ANOVA – Semantic × group interaction
A
B

## Slide 12
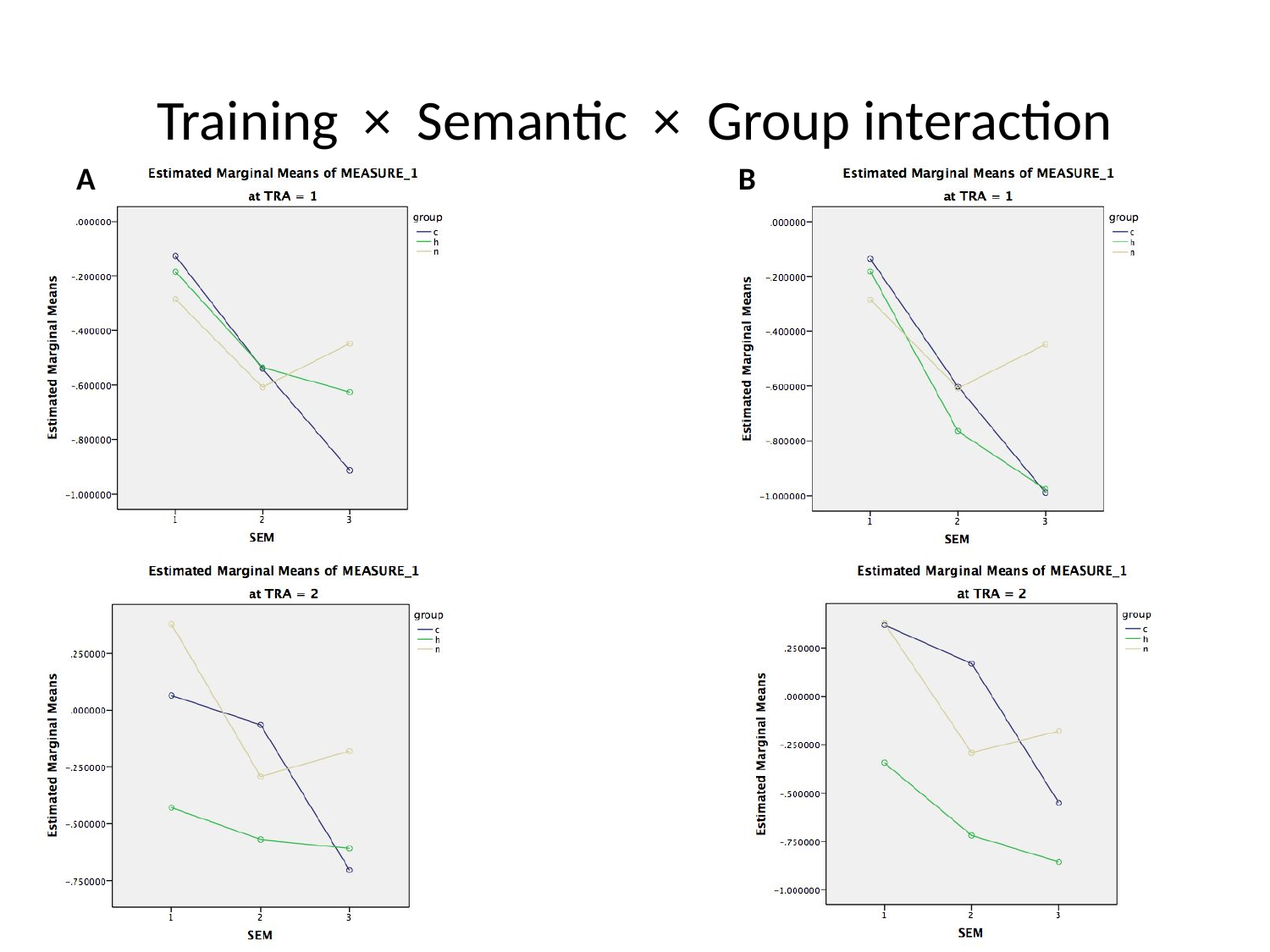

# Training × Semantic × Group interaction
A
B

## Slide 13
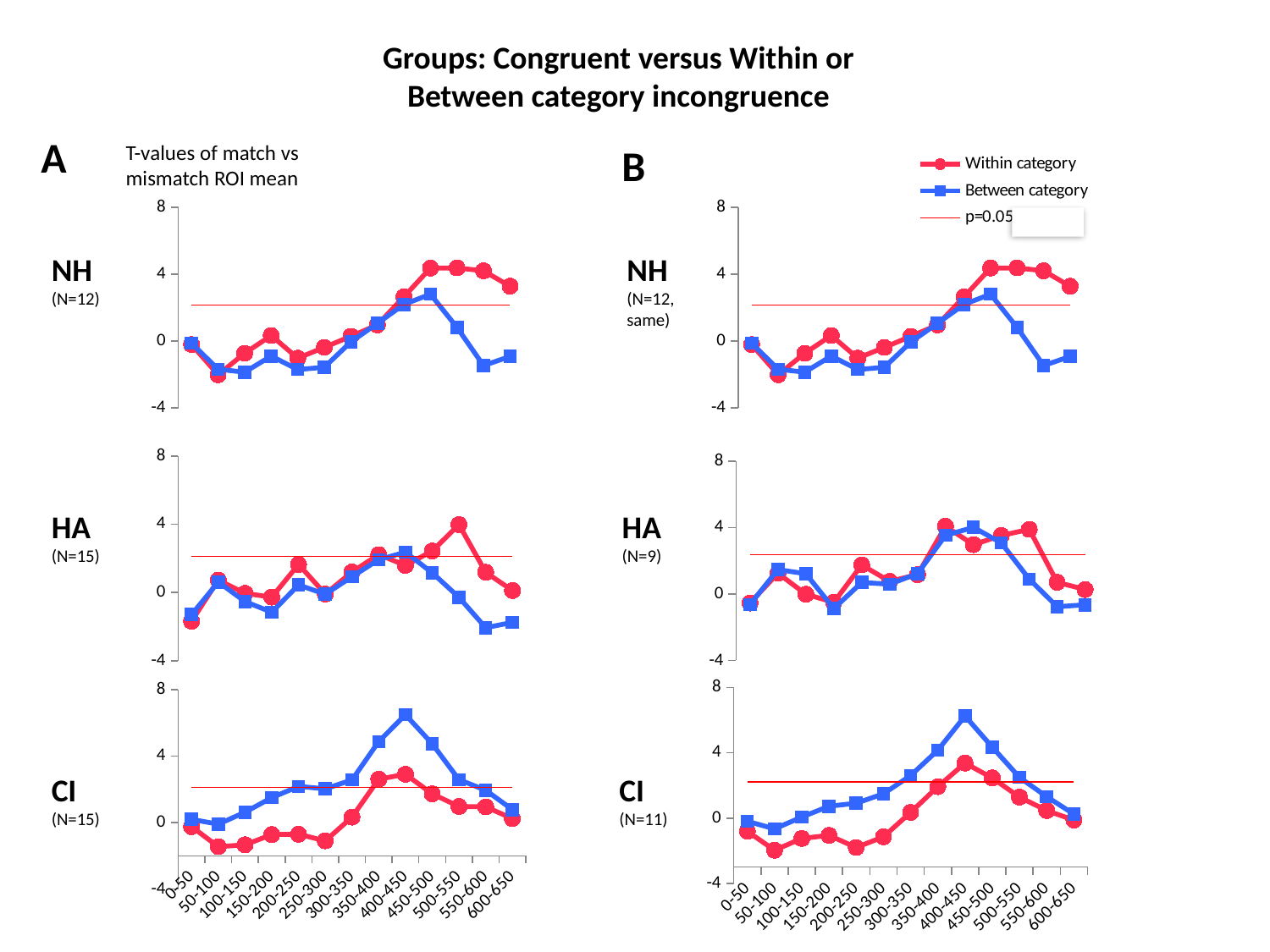

### Chart
| Category | Within category | Between category | p=0.05 (N=12) |
|---|---|---|---|
| 0-50 | -0.204 | -0.128 | 2.18 |
| 50-100 | -2.011 | -1.681 | 2.18 |
| 100-150 | -0.724 | -1.855 | 2.18 |
| 150-200 | 0.335 | -0.891 | 2.18 |
| 200-250 | -1.018 | -1.699 | 2.18 |
| 250-300 | -0.373 | -1.561 | 2.18 |
| 300-350 | 0.288 | -0.048 | 2.18 |
| 350-400 | 0.971 | 1.063 | 2.18 |
| 400-450 | 2.65 | 2.196 | 2.18 |
| 450-500 | 4.371 | 2.809 | 2.18 |
| 500-550 | 4.381 | 0.82 | 2.18 |
| 550-600 | 4.207 | -1.46 | 2.18 |
| 600-650 | 3.285 | -0.915 | 2.18 |
### Chart
| Category | Within category | Between category | p=0.05 (N=12) |
|---|---|---|---|
| 0-50 | -0.204 | -0.128 | 2.18 |
| 50-100 | -2.011 | -1.681 | 2.18 |
| 100-150 | -0.724 | -1.855 | 2.18 |
| 150-200 | 0.335 | -0.891 | 2.18 |
| 200-250 | -1.018 | -1.699 | 2.18 |
| 250-300 | -0.373 | -1.561 | 2.18 |
| 300-350 | 0.288 | -0.048 | 2.18 |
| 350-400 | 0.971 | 1.063 | 2.18 |
| 400-450 | 2.65 | 2.196 | 2.18 |
| 450-500 | 4.371 | 2.809 | 2.18 |
| 500-550 | 4.381 | 0.82 | 2.18 |
| 550-600 | 4.207 | -1.46 | 2.18 |
| 600-650 | 3.285 | -0.915 | 2.18 |Groups: Congruent versus Within or Between category incongruence
A
T-values of match vs mismatch ROI mean
B
NH
(N=12)
NH
(N=12,
same)
### Chart
| Category | Within category | Between category | p=0.05 (N=15) |
|---|---|---|---|
| 0-50 | -1.688 | -1.301 | 2.13 |
| 50-100 | 0.727 | 0.61 | 2.13 |
| 100-150 | -0.054 | -0.538 | 2.13 |
| 150-200 | -0.276 | -1.161 | 2.13 |
| 200-250 | 1.641 | 0.441 | 2.13 |
| 250-300 | -0.095 | -0.107 | 2.13 |
| 300-350 | 1.196 | 0.921 | 2.13 |
| 350-400 | 2.218 | 1.92 | 2.13 |
| 400-450 | 1.586 | 2.354999999999999 | 2.13 |
| 450-500 | 2.427 | 1.151 | 2.13 |
| 500-550 | 3.977 | -0.289 | 2.13 |
| 550-600 | 1.179 | -2.07 | 2.13 |
| 600-650 | 0.109 | -1.756 | 2.13 |
### Chart
| Category | Within category | Between category | p=0.05 (N=9) |
|---|---|---|---|
| 0-50 | -0.543 | -0.631 | 2.36 |
| 50-100 | 1.254 | 1.472 | 2.36 |
| 100-150 | -0.011 | 1.219 | 2.36 |
| 150-200 | -0.505 | -0.877 | 2.36 |
| 200-250 | 1.752 | 0.697 | 2.36 |
| 250-300 | 0.762 | 0.592 | 2.36 |
| 300-350 | 1.168 | 1.233 | 2.36 |
| 350-400 | 4.082 | 3.534 | 2.36 |
| 400-450 | 2.976 | 4.018 | 2.36 |
| 450-500 | 3.526 | 3.082 | 2.36 |
| 500-550 | 3.9 | 0.897 | 2.36 |
| 550-600 | 0.706 | -0.767 | 2.36 |
| 600-650 | 0.272 | -0.649 | 2.36 |
HA
(N=15)
HA
(N=9)
### Chart
| Category | Within category | Between category | p=0.05 (N=15) |
|---|---|---|---|
| 0-50 | -0.247 | 0.196 | 2.13 |
| 50-100 | -1.452 | -0.099 | 2.13 |
| 100-150 | -1.337 | 0.624 | 2.13 |
| 150-200 | -0.722 | 1.5 | 2.13 |
| 200-250 | -0.701 | 2.175 | 2.13 |
| 250-300 | -1.101 | 2.03 | 2.13 |
| 300-350 | 0.327 | 2.564 | 2.13 |
| 350-400 | 2.612 | 4.875 | 2.13 |
| 400-450 | 2.908 | 6.49 | 2.13 |
| 450-500 | 1.729 | 4.744 | 2.13 |
| 500-550 | 0.966 | 2.584 | 2.13 |
| 550-600 | 0.944 | 1.944 | 2.13 |
| 600-650 | 0.24 | 0.779 | 2.13 |
### Chart
| Category | Within category | Between category | P=0.05 (N=11) |
|---|---|---|---|
| 0-50 | -0.806 | -0.198 | 2.22 |
| 50-100 | -1.968 | -0.66 | 2.22 |
| 100-150 | -1.246 | 0.062 | 2.22 |
| 150-200 | -1.056 | 0.73 | 2.22 |
| 200-250 | -1.8 | 0.912 | 2.22 |
| 250-300 | -1.146 | 1.49 | 2.22 |
| 300-350 | 0.352 | 2.606 | 2.22 |
| 350-400 | 1.925 | 4.156 | 2.22 |
| 400-450 | 3.37 | 6.253 | 2.22 |
| 450-500 | 2.46 | 4.348 | 2.22 |
| 500-550 | 1.289 | 2.494 | 2.22 |
| 550-600 | 0.463 | 1.314 | 2.22 |
| 600-650 | -0.123 | 0.246 | 2.22 |CI
(N=15)
CI
(N=11)

## Slide 14
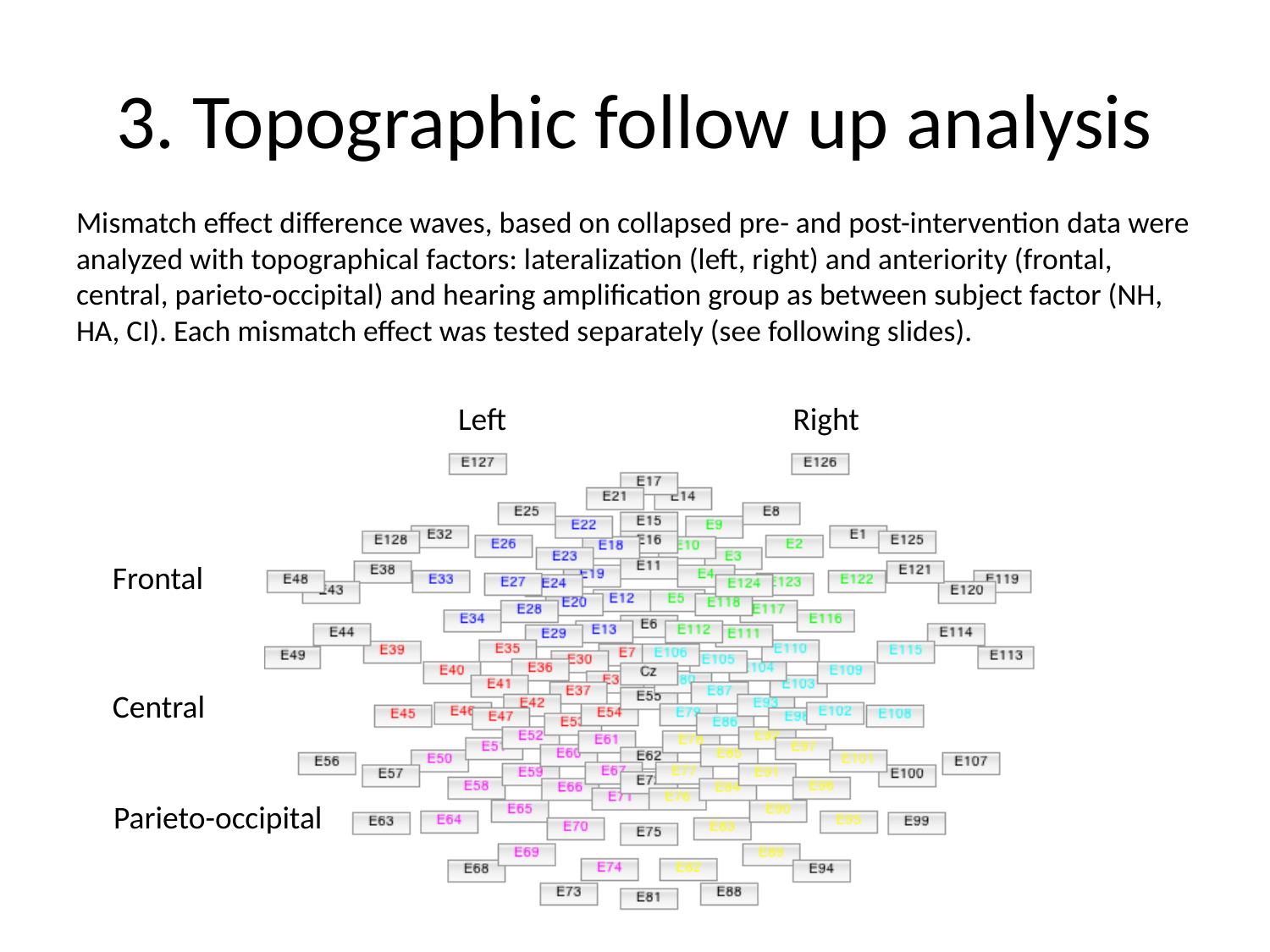

# 3. Topographic follow up analysis
Mismatch effect difference waves, based on collapsed pre- and post-intervention data were analyzed with topographical factors: lateralization (left, right) and anteriority (frontal, central, parieto-occipital) and hearing amplification group as between subject factor (NH, HA, CI). Each mismatch effect was tested separately (see following slides).
Left
Right
Frontal
Central
Parieto-occipital

## Slide 15
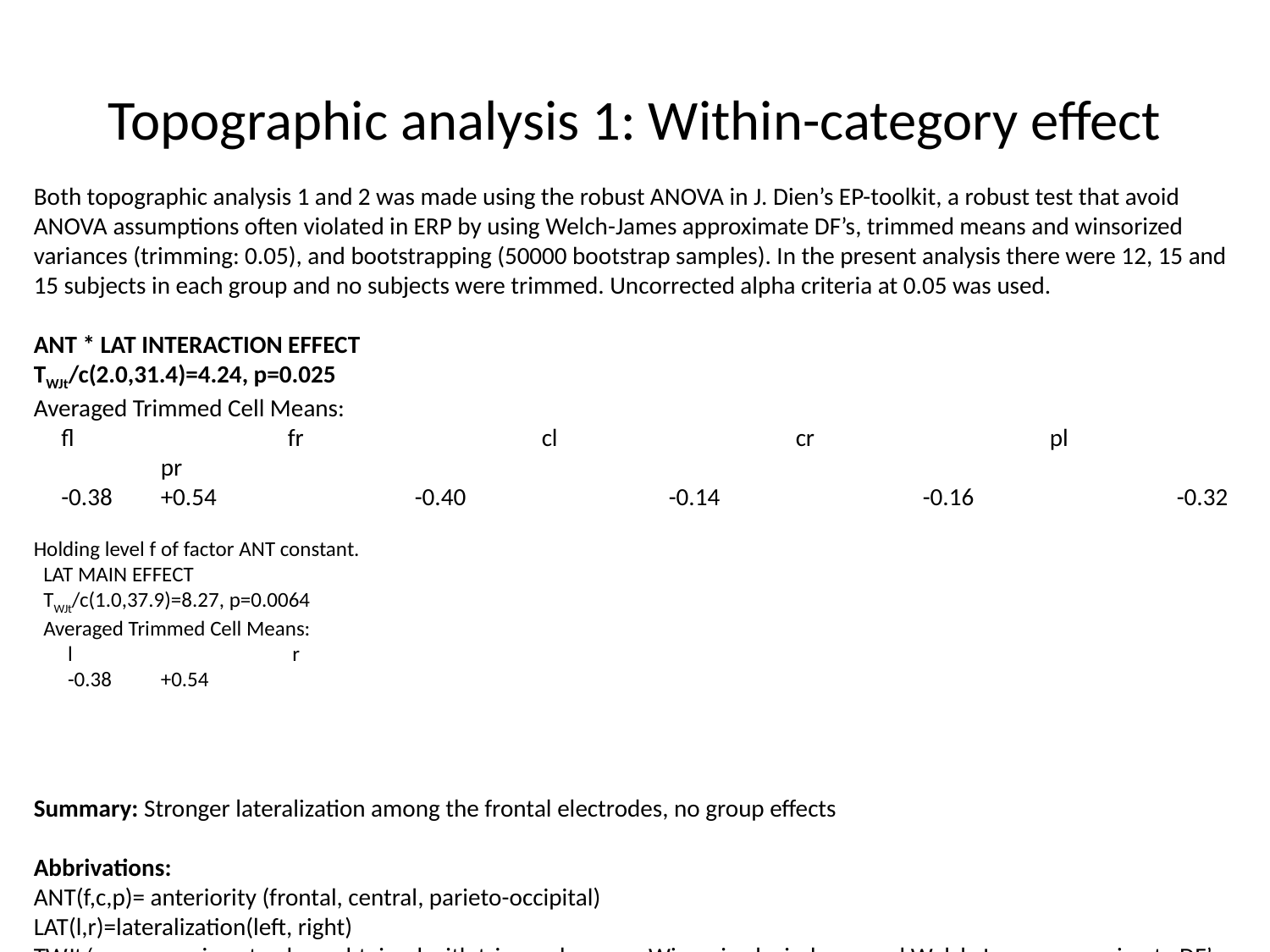

# Topographic analysis 1: Within-category effect
Both topographic analysis 1 and 2 was made using the robust ANOVA in J. Dien’s EP-toolkit, a robust test that avoid ANOVA assumptions often violated in ERP by using Welch-James approximate DF’s, trimmed means and winsorized variances (trimming: 0.05), and bootstrapping (50000 bootstrap samples). In the present analysis there were 12, 15 and 15 subjects in each group and no subjects were trimmed. Uncorrected alpha criteria at 0.05 was used.
ANT * LAT INTERACTION EFFECT
TWJt/c(2.0,31.4)=4.24, p=0.025
Averaged Trimmed Cell Means:
     fl		fr		cl   		cr   		pl   		pr
     -0.38	+0.54		-0.40		-0.14		-0.16		-0.32
Holding level f of factor ANT constant.
  LAT MAIN EFFECT
  TWJt/c(1.0,37.9)=8.27, p=0.0064
  Averaged Trimmed Cell Means:
       l   		 r
       -0.38	+0.54
Summary: Stronger lateralization among the frontal electrodes, no group effects
Abbrivations:
ANT(f,c,p)= anteriority (frontal, central, parieto-occipital)
LAT(l,r)=lateralization(left, right)
TWJt/c = comparison t-values obtained with trimmed means, Winsorized windows, and Welch-James approximate DF’s.

## Slide 16
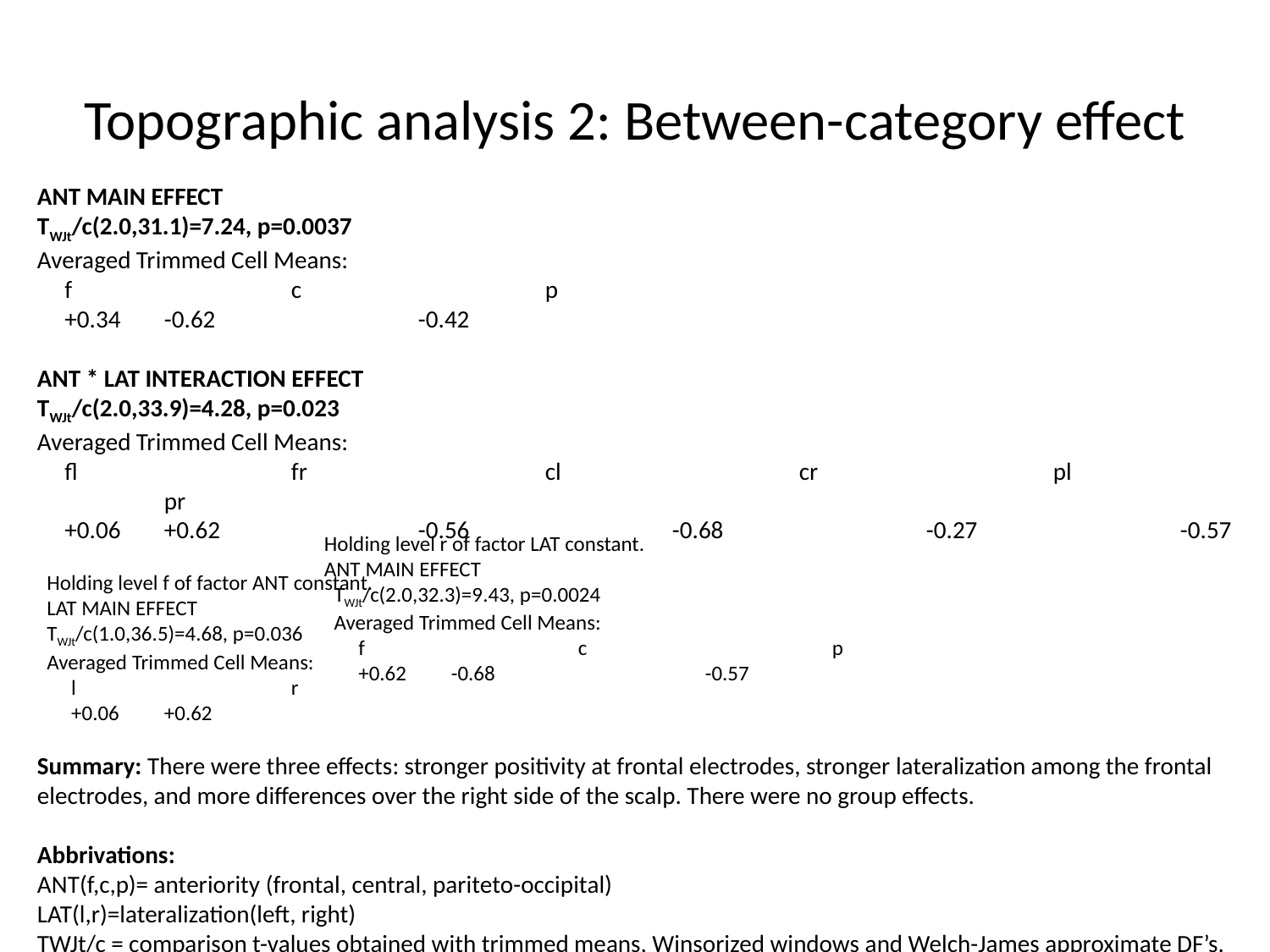

# Topographic analysis 2: Between-category effect
ANT MAIN EFFECT
TWJt/c(2.0,31.1)=7.24, p=0.0037
Averaged Trimmed Cell Means:
     f    		c    		p
     +0.34	-0.62		-0.42
ANT * LAT INTERACTION EFFECT
TWJt/c(2.0,33.9)=4.28, p=0.023
Averaged Trimmed Cell Means:
     fl   		fr		cl   		cr  		pl   		pr
     +0.06	+0.62		-0.56		-0.68		-0.27		-0.57
  Holding level f of factor ANT constant.
  LAT MAIN EFFECT
  TWJt/c(1.0,36.5)=4.68, p=0.036
  Averaged Trimmed Cell Means:
       l		r
       +0.06	+0.62
Summary: There were three effects: stronger positivity at frontal electrodes, stronger lateralization among the frontal electrodes, and more differences over the right side of the scalp. There were no group effects.
Abbrivations:
ANT(f,c,p)= anteriority (frontal, central, pariteto-occipital)
LAT(l,r)=lateralization(left, right)
TWJt/c = comparison t-values obtained with trimmed means, Winsorized windows and Welch-James approximate DF’s.
Holding level r of factor LAT constant.
ANT MAIN EFFECT
  TWJt/c(2.0,32.3)=9.43, p=0.0024
  Averaged Trimmed Cell Means:
       f		c		p
       +0.62	-0.68		-0.57

## Slide 17
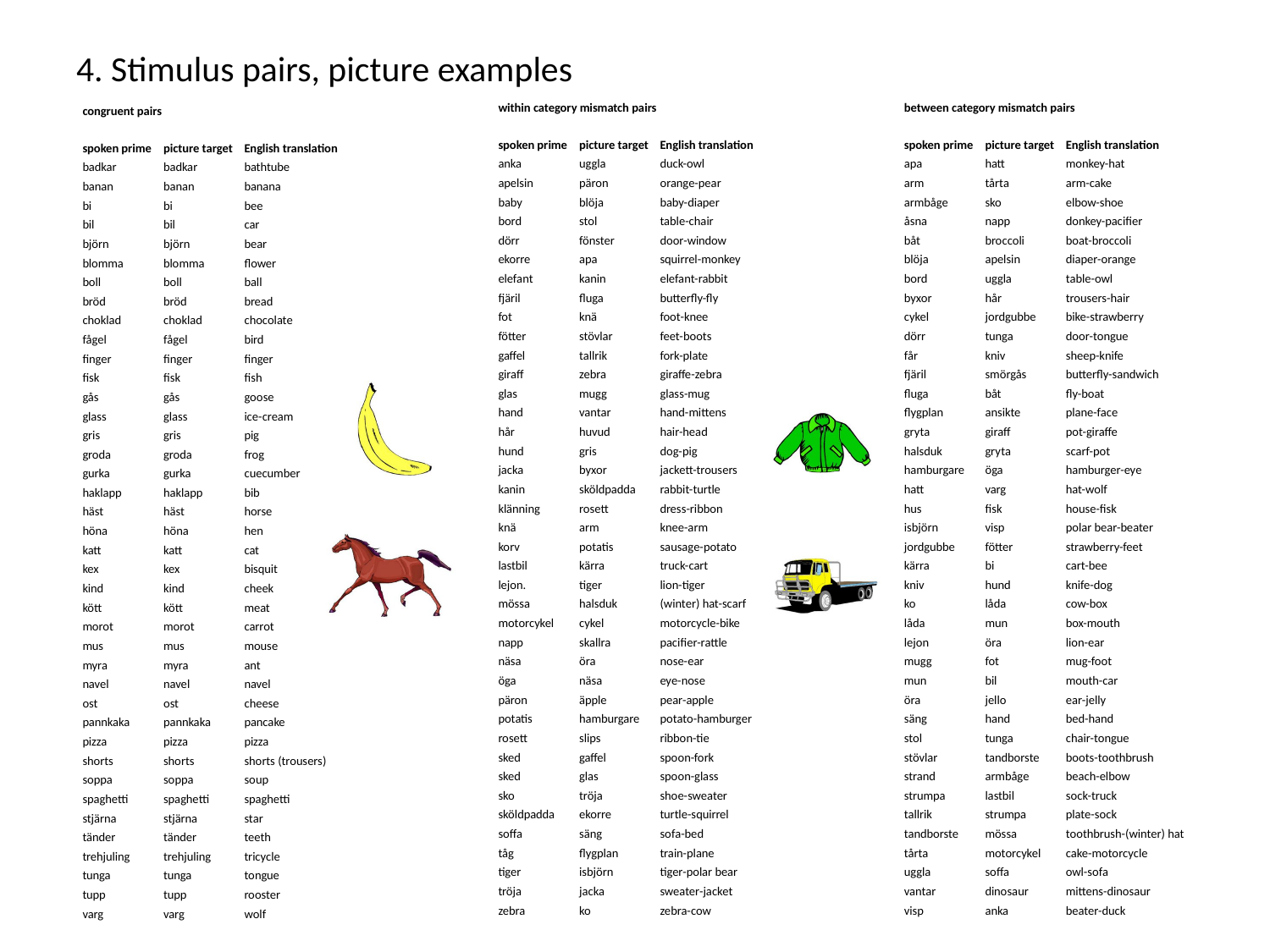

# 4. Stimulus pairs, picture examples
| between category mismatch pairs | | |
| --- | --- | --- |
| spoken prime | picture target | English translation |
| apa | hatt | monkey-hat |
| arm | tårta | arm-cake |
| armbåge | sko | elbow-shoe |
| åsna | napp | donkey-pacifier |
| båt | broccoli | boat-broccoli |
| blöja | apelsin | diaper-orange |
| bord | uggla | table-owl |
| byxor | hår | trousers-hair |
| cykel | jordgubbe | bike-strawberry |
| dörr | tunga | door-tongue |
| får | kniv | sheep-knife |
| fjäril | smörgås | butterfly-sandwich |
| fluga | båt | fly-boat |
| flygplan | ansikte | plane-face |
| gryta | giraff | pot-giraffe |
| halsduk | gryta | scarf-pot |
| hamburgare | öga | hamburger-eye |
| hatt | varg | hat-wolf |
| hus | fisk | house-fisk |
| isbjörn | visp | polar bear-beater |
| jordgubbe | fötter | strawberry-feet |
| kärra | bi | cart-bee |
| kniv | hund | knife-dog |
| ko | låda | cow-box |
| låda | mun | box-mouth |
| lejon | öra | lion-ear |
| mugg | fot | mug-foot |
| mun | bil | mouth-car |
| öra | jello | ear-jelly |
| säng | hand | bed-hand |
| stol | tunga | chair-tongue |
| stövlar | tandborste | boots-toothbrush |
| strand | armbåge | beach-elbow |
| strumpa | lastbil | sock-truck |
| tallrik | strumpa | plate-sock |
| tandborste | mössa | toothbrush-(winter) hat |
| tårta | motorcykel | cake-motorcycle |
| uggla | soffa | owl-sofa |
| vantar | dinosaur | mittens-dinosaur |
| visp | anka | beater-duck |
| within category mismatch pairs | | |
| --- | --- | --- |
| spoken prime | picture target | English translation |
| anka | uggla | duck-owl |
| apelsin | päron | orange-pear |
| baby | blöja | baby-diaper |
| bord | stol | table-chair |
| dörr | fönster | door-window |
| ekorre | apa | squirrel-monkey |
| elefant | kanin | elefant-rabbit |
| fjäril | fluga | butterfly-fly |
| fot | knä | foot-knee |
| fötter | stövlar | feet-boots |
| gaffel | tallrik | fork-plate |
| giraff | zebra | giraffe-zebra |
| glas | mugg | glass-mug |
| hand | vantar | hand-mittens |
| hår | huvud | hair-head |
| hund | gris | dog-pig |
| jacka | byxor | jackett-trousers |
| kanin | sköldpadda | rabbit-turtle |
| klänning | rosett | dress-ribbon |
| knä | arm | knee-arm |
| korv | potatis | sausage-potato |
| lastbil | kärra | truck-cart |
| lejon. | tiger | lion-tiger |
| mössa | halsduk | (winter) hat-scarf |
| motorcykel | cykel | motorcycle-bike |
| napp | skallra | pacifier-rattle |
| näsa | öra | nose-ear |
| öga | näsa | eye-nose |
| päron | äpple | pear-apple |
| potatis | hamburgare | potato-hamburger |
| rosett | slips | ribbon-tie |
| sked | gaffel | spoon-fork |
| sked | glas | spoon-glass |
| sko | tröja | shoe-sweater |
| sköldpadda | ekorre | turtle-squirrel |
| soffa | säng | sofa-bed |
| tåg | flygplan | train-plane |
| tiger | isbjörn | tiger-polar bear |
| tröja | jacka | sweater-jacket |
| zebra | ko | zebra-cow |
| congruent pairs | | |
| --- | --- | --- |
| spoken prime | picture target | English translation |
| badkar | badkar | bathtube |
| banan | banan | banana |
| bi | bi | bee |
| bil | bil | car |
| björn | björn | bear |
| blomma | blomma | flower |
| boll | boll | ball |
| bröd | bröd | bread |
| choklad | choklad | chocolate |
| fågel | fågel | bird |
| finger | finger | finger |
| fisk | fisk | fish |
| gås | gås | goose |
| glass | glass | ice-cream |
| gris | gris | pig |
| groda | groda | frog |
| gurka | gurka | cuecumber |
| haklapp | haklapp | bib |
| häst | häst | horse |
| höna | höna | hen |
| katt | katt | cat |
| kex | kex | bisquit |
| kind | kind | cheek |
| kött | kött | meat |
| morot | morot | carrot |
| mus | mus | mouse |
| myra | myra | ant |
| navel | navel | navel |
| ost | ost | cheese |
| pannkaka | pannkaka | pancake |
| pizza | pizza | pizza |
| shorts | shorts | shorts (trousers) |
| soppa | soppa | soup |
| spaghetti | spaghetti | spaghetti |
| stjärna | stjärna | star |
| tänder | tänder | teeth |
| trehjuling | trehjuling | tricycle |
| tunga | tunga | tongue |
| tupp | tupp | rooster |
| varg | varg | wolf |

## Slide 18
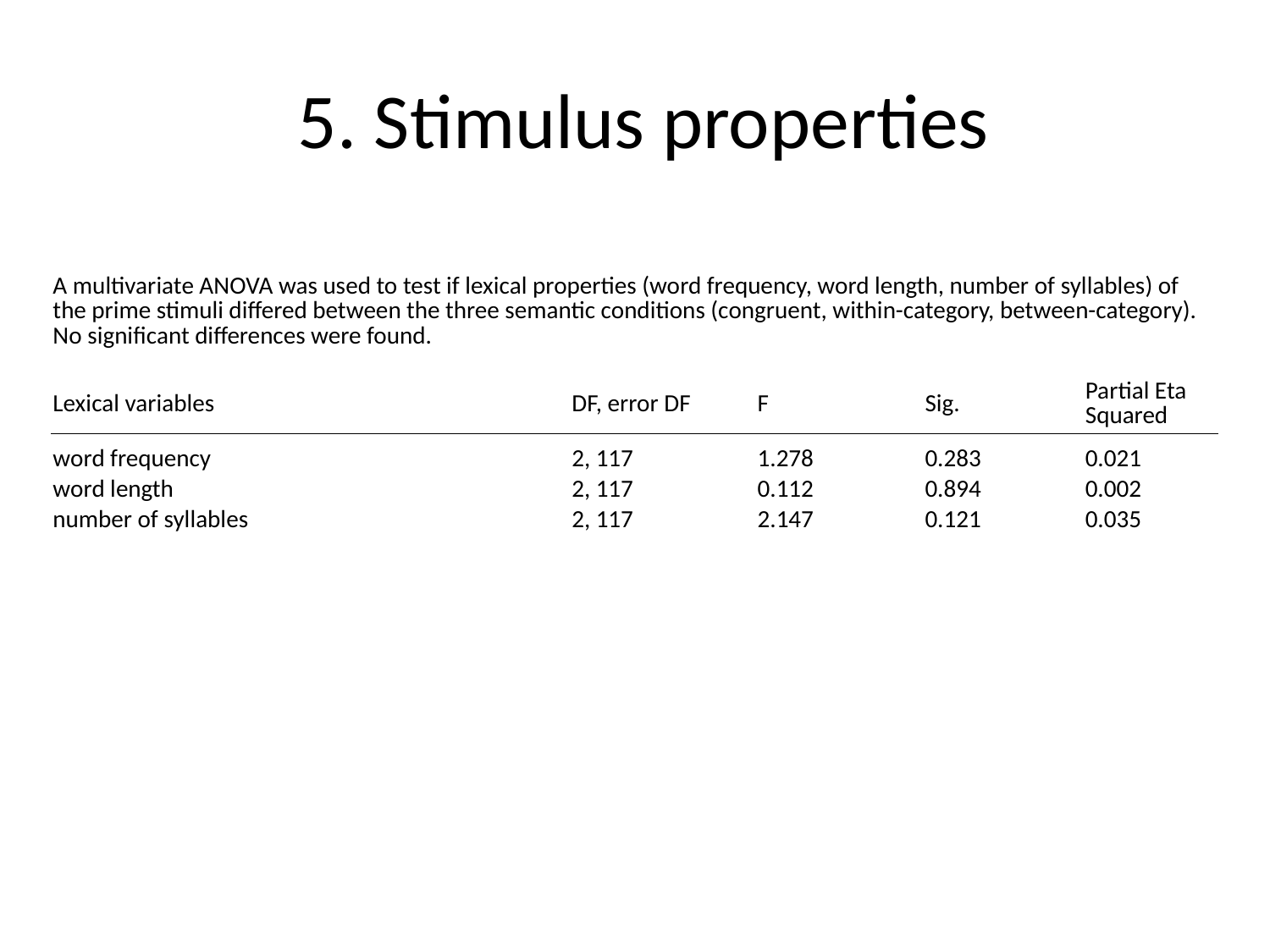

# 5. Stimulus properties
| A multivariate ANOVA was used to test if lexical properties (word frequency, word length, number of syllables) of the prime stimuli differed between the three semantic conditions (congruent, within-category, between-category). No significant differences were found. | | | | |
| --- | --- | --- | --- | --- |
| Lexical variables | DF, error DF | F | Sig. | Partial Eta Squared |
| word frequency | 2, 117 | 1.278 | 0.283 | 0.021 |
| word length | 2, 117 | 0.112 | 0.894 | 0.002 |
| number of syllables | 2, 117 | 2.147 | 0.121 | 0.035 |
